# A novel mechanism behind aerial dispersal of pycnidiospores

**DOI:** 10.1128/msphere.00034-26

**Published:** 2026-06-10

**Authors:** Satyendra Pratap Singh, Alon Shomron, Ran Shulhani, Dani Shtienberg, Noam Alkan

**Affiliations:** 1 Department of Postharvest Science, ARO, Volcani Institute 530612 , Rishon LeZiyyon, Israel; 2 Faculty of Agriculture, Food and Environment, The Hebrew University of Jerusalem 72256, Rehovot, Israel; 3 Department of Plant Pathology and Weed Science, ARO, Volcani Institute 530612 , Rishon LeZiyyon, Israel; Ben-Gurion University of the Negev, Beer-Sheva, Israel

**Keywords:** pycnidiospores dispersal, relative humidity, pycnidial burst, fungal spores, aerial dispersal, disease epidemiology

## Abstract

Pycnidia are asexual fruiting bodies formed by diverse Ascomycete fungi, comprising many destructive horticultural pathogens within Diaporthales, Pleosporales, and Botryosphaeriales. These fungi disperse over long distances via airborne sexual ascospores, while their asexual pycnidiospores are thought to spread locally, mainly by water splash. However, in *Botryosphaeriaceae*, including *Lasiodiplodia theobromae*, the sexual stage is rarely observed, never reported in Israel, yet disease exhibits frequent long-distance aerial dispersal of pycnidiospores. This research aimed to elucidate the mechanisms underlying pycnidial burst and aerial dispersal of pycnidiospores, using *L. theobromae* as a model. Field observations correlated new infections with seasonal relative humidity (RH). *In vitro* findings and scanning electron micrograph confirmed that high RH triggers pycnidial bursts, while aerial pycnidiospore dispersal follows subsequent RH decline. Wind tunnel experiments revealed that pycnidiospores were released at RH >70%, and their travel distances increased with wind speed (2.0 m s^−1^), with spores detected up to the tunnel’s end. The pycnidial analysis revealed high osmolyte concentrations (~0.17 M; sugars and glycerol), generating internal pressure of 4.18 atmospheres. These findings support a new mechanism for pycnidiospore release: under high RH, moisture permeates the closed pycnidia, dissolves osmolytes, and increases osmotic pressure to trigger pycnidial bursts. As RH drops, the mucilage surrounding the pycnidiospores dries, enabling the pycnidiospores to disperse by the wind over long distances. This mechanism was observed across *Botryosphaeriaceae* species infecting different trees. These results provide an overlooked mechanism for long-distance dispersal of pycnidiospores mediated by humidity-driven pycnidial burst and wind-assisted transport, with implications for disease epidemiology and management in agricultural systems.

**IMPORTANCE:**

Airborne dispersal of fungal plant pathogens is generally attributed to sexual spores, whereas asexual pycnidiospores are widely assumed to spread locally via water splash. This assumption is difficult to reconcile with frequent long-distance disease outbreaks caused by fungi, that is, *Botryosphaeriaceae*, in which the sexual stage is rare or absent, including *Lasiodiplodia theobromae*. Here, we demonstrate that pycnidiospores can disperse aerially over long distances under rainless conditions through a previously unrecognized mechanism. We show that high relative humidity induces pressurized pycnidial bursts driven by osmolytes, including sugars and glycerol, generating sufficient internal pressure to rupture pycnidia. As humidity subsequently declines, released pycnidiospores dry and become wind-dispersible. This mechanism explains how asexual pycnidiospores of various species can contribute to long-distance disease spread and challenges long-standing assumptions about fungal dispersal ecology. Our findings have broad implications for understanding fungal epidemiology and improving disease forecasting and management in agricultural systems.

## INTRODUCTION

The rising incidence of fungal plant disease outbreaks poses substantial and increasing risks to agricultural productivity and global food security. These outbreaks affect both managed and natural ecosystems, cause significant yield losses, and emerge in new geographical areas (1, 2). Fungal pathogens are well documented for spreading into new territories, with historical and current examples across forest and cultivated plant systems (3). The emergence of many plant disease epidemics often hinges on the dispersal of fungal spores, which can initiate outbreaks in host plantations and forests (4).

Fungal pathogens may reproduce via both sexual and asexual spores, which differ in their production mechanisms and dispersal strategies. Sexual spores such as ascospores (Ascomycota), basidiospores (Basidiomycota), oospores (Oomycota), and zygospores (Zygomycota) are produced by the teleomorph stage. These spores can travel long distances through the air by wind (5–8). Environmental factors such as rainfall, relative humidity (RH), temperature, and wind play a significant role in regulating the release and dispersal of fungal spores from the various fruiting bodies (4, 5, 9–12). In contrast, asexual spores are produced during the anamorph stage, develop in a variety of conidiogenous structures, including exposed conidiophores, acervuli, sporangia (Zygomycota), or pycnidia (Ascomycota). Some asexual conidia can also be spread through the air under certain environmental conditions (5). Asexual spores formed within a pycnidium are typically known to disperse over shorter distances through water splashes caused by raindrops or agricultural watering regimes (13–16).

The *Botryosphaeriaceae* family, within the Ascomycota phylum, infects a wide range of woody plants, accounting for some of the most devastating fungal phytopathogens, including *Lasiodiplodia theobromae, Neoscytalidium dimidiatum, Neofusicoccum parvum, Diplodia neojuniperi, Dethiorellla viticola,* etc. (17, 18). Among them, *L. theobromae* is one of the most aggressive pathogens of this group, infecting a diverse range of vascular plants in agricultural settings, including avocado (19), mango (20), grapevine (21), cocoa (22), cannabis (23), as well as pine, eucalyptus, and oak in both natural forests and managed plantations. The disease symptoms associated with *L. theobromae* include cankers on tree trunks, dieback, collar rot, and leaf blight (22). Moreover, the agricultural losses associated with *L. theobromae* extend into the postharvest via stem end rot of fruit (24, 25).

Fungal pathogens from the *Botryosphaeriaceae* family produce sexual (ascomata) and asexual (pycnidia) fruiting bodies. Ascomata forcefully expel sexual spores, specifically ascospores, which are known for their long-range dissemination via air currents (5, 26). However, direct field observations of sexual reproduction and ascospore release in *L. theobromae* and related *Botryosphaeriaceae* are reported only sporadically (27), and such events have not been observed in Israel (28). In contrast, for rapid reproduction, asexual pycnidiospores are produced and discharged from pycnidia that are formed on the exterior of infected branches (13, 14, 29, 30). These pycnidiospores are considered to regularly disperse over short distances, preliminarily via rain splash or water movement (13, 14, 29). Nevertheless, some studies also have documented the occurrence of pycnidiospores in the air during rainless periods in different pycnidial fungi, indicating that aerial dispersal of asexual spores can occur under specific environmental conditions (e.g., RH, wind) and overhead irrigation (21, 28, 31).

**Fig 1:**
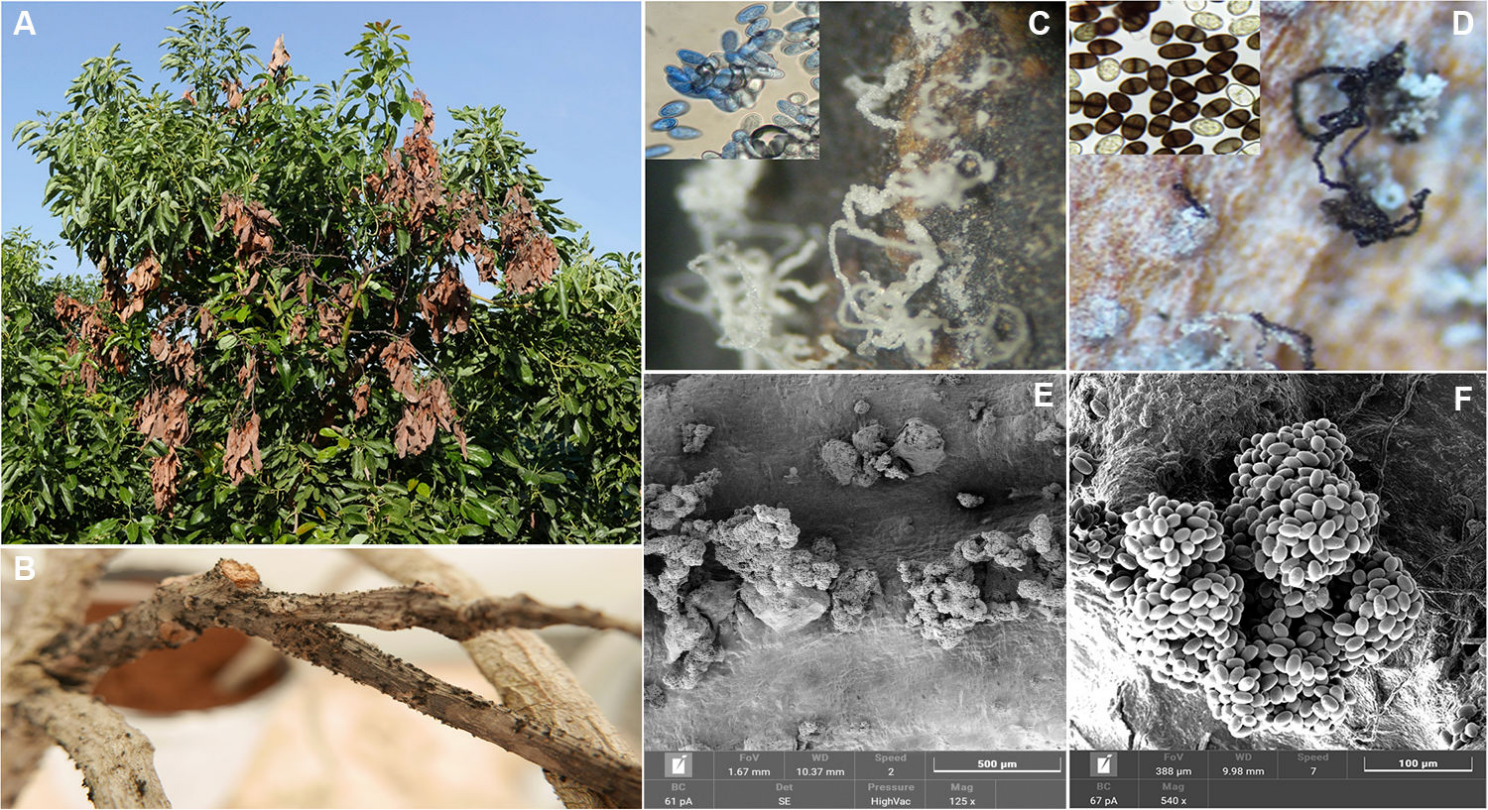
*Botryosphaeriaceae* dieback symptoms and pycnidiospores released from plant stems. (**A**) Dieback symptoms caused by *L. theobromae* in the upper canopy of an avocado tree; (**B**) infected avocado twig grown in the greenhouse with dried cirrhi secreted from pycnidia; (**C and D**) binocular pictures of the burst dried cirrhi secreted from pycnidia in the greenhouse containing immature and mature pycnidiospores. Inner photos: individual pycnidiospores; (**E and F**) SEMs of the release of pycnidiospores after pycnidial burst.

Although considerable knowledge has been accumulated on *Botryosphaeriaceae*-associated diseases (32–34), this information has not been integrated into a unified paradigm for pycnidial burst and long-distance aerial dispersal of pycnidiospores. Several field observations have been made in the avocado orchards in Israel, revealing sporadic dieback that developed simultaneously on the upper branches and inflorescence of the trees (Fig. 1A). Further investigation confirms that these symptoms were caused by the *Botryosphaeriaceae* pathogen *L. theobromae*. The sporadic, patchy, and scattered occurrence of dieback in the trees suggested that it resulted from aerial dispersion of *L. theobromae* and initially presumed that the infections resulted from ascospore dissemination. To examine this possibility, we looked for the sexual fruiting bodies of the fungi (ascomata), which produce ascospores that are known to be dispersed by air. Despite extensive searches over a decade, no sexual fruiting bodies of *Botryosphaeriaceae* were detected in Israel’s orchards and natural forests. This finding aligns with global reports indicating the rare occurrence of ascomata of *Botryosphaeriaceae* in nature. At the same time, many asexual fruiting bodies (pycnidia) of *Lasiodiplodia* are commonly found on infected tree branches in Israel (28) and elsewhere (14, 35), which seem to disperse by air.

To investigate this phenomenon, greenhouse trials were conducted under ambient environmental conditions, where *L. theobromae* was inoculated in avocado tree branches protected from rain. Despite protection from rain, typical dieback symptoms had developed on the inoculated trees after 1 to 2 weeks. Surprisingly, the non-inoculated control trees located in the same greenhouse developed dieback symptoms, likely due to unintentional exposure to *L. theobromae* from nearby inoculated trees. Observations showed that pycnidiospores were secreted from pycnidia developing on the inoculated trees and appeared on the outer surface of the stems (Fig. 1C through F), suggesting they could potentially be dispersed by wind even in the absence of rainfall.

Based on the abovementioned observations and previous reports, we hypothesize that pycnidiospores can be released from pycnidia under defined meteorological conditions, rainless days, and be dispersed aerially. This study employs *L. theobromae* and avocado as a model system to investigate the mechanisms underlying pycnidial burst and the environmental triggers enabling long-distance aerial dissemination of pycnidiospores, with implications for disease dynamics in agricultural and natural ecosystems.

## RESULTS

### Multiple *Botryosphaeriaceae* pathogens exhibited consistent patterns of pycnidial burst across agricultural and forest plantations

**Fig 2:**
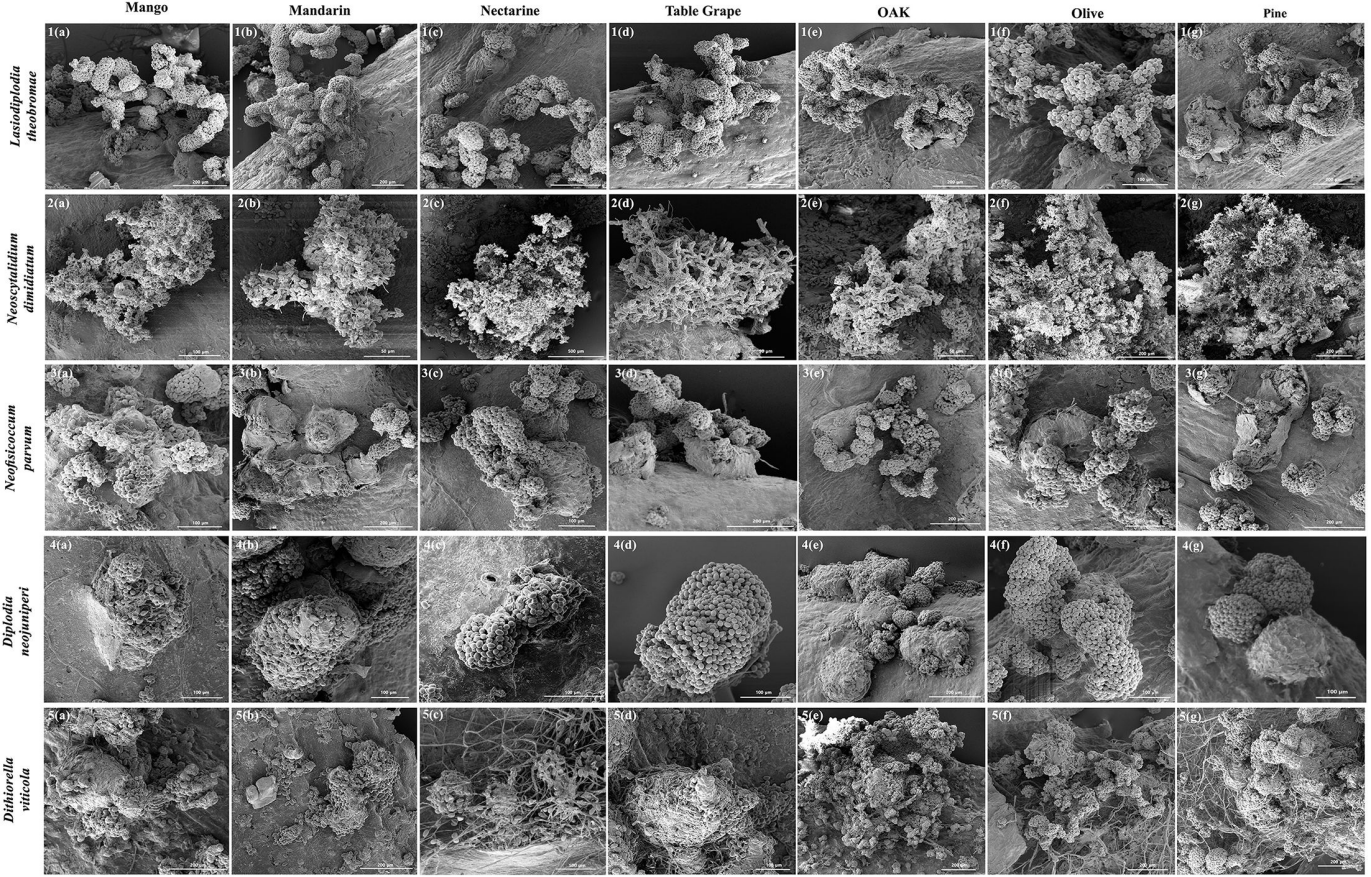
Pycnidiospore release patterns of different pathogens of *Botryosphaeriaceae* in different plant hosts. SEMs depict the release of pycnidiospores of *Lasiodiplodia theobromae* (1)*, Neoscytalidium dimidiatum* (2)*, Neofusicoccum parvum* (3)*, Diplodia neojuniperi* (4)*,* and *Dethiorella viticola* (5) from the infected twigs of agricultural and forest trees (mango, mandarin, nectarine, table grapes, oak, olive, and pine).

Scanning electron micrographs (SEMs) of pycnidial bursts in plant twigs from various agricultural and natural forest plantations infected with various species of *Botryosphaeriaceae* pathogens, including *L. theobromae* (B-43)*, N. dimidiatum* (B-47)*, N. parvum* (B-33)*, D. neojuniperi* (B-37)*,* and *D. viticola* (B-38) (Fig. 2). After incubation at high humidity, pycnidial formations began to emerge on the twig surfaces, subsequently rupturing the pycnidia and releasing the pycnidiospores. The results indicated that, although each *Botryosphaeriaceae* pathogen has a specific pattern of pycnidiospore release, the overall mechanism involving pycnidia rupture at high humidity remained consistent across all examined pathogens and all examined twigs from different trees (Fig. 2).

### Annual trend of *Botryosphaeriaceae* colonization in young avocado twigs

**Fig 3:**
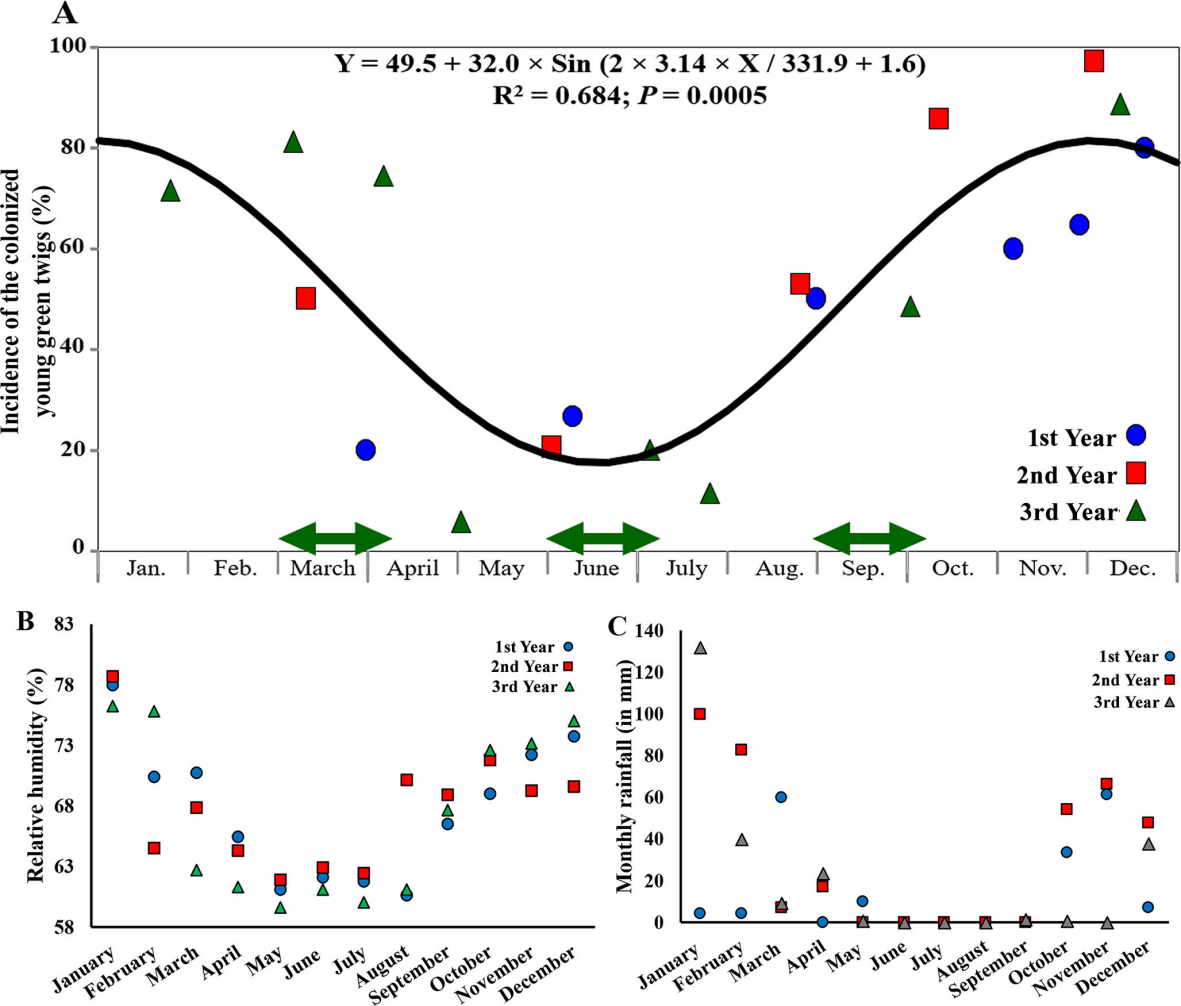
Colonization of *Botryosphaeria* in green, young twigs of avocado trees over 3 consecutive years. (**A**) Yearly changes in the incidence of colonization in young green twigs; (**B**) monthly average of relative humidity (%); and (**C**) cumulative monthly rain (mm) of the respective years.

The colonization of young, green twigs of avocado trees by different species of *Botryosphaeriaceae,* including *L. theobromae, Neofusicoccum parvum, Botryosphaeria dothidea, Neoscytalidium dimidiatum, Diplodia viticola,* and *Diplodia neojuniperi,* was recorded over 3 consecutive years (2014 to 2016) to assess the seasonal infection dynamics. Data analysis indicated an annual trend in *Botryosphaeriaceae* colonization; among them, *Lasiodiplodia* was the majority of infections. A higher colonization incidence was observed in the autumn and winter (October to March), with a cumulative incidence of 71.3% (range: 65%–79.8%), compared to spring and summer (May to July), which recorded a cumulative incidence of 16.9% (range: 5.7%–26.7%) (Fig. 3A). Corresponding monthly average RH (%) and cumulative rainfall (mm) data of the respective years also exhibited a similar pattern, with high RH and rainfall during autumn and winter gradually decreasing during hot summers. It should be noted that the first rainfall events in the 3 years of sampling occurred in autumn (October), yet an increase in colonization incidence was observed in mid-summer (July), in periods of very little rain. Despite variations in rainfall patterns, such as delayed onset in December 2016 and minimal precipitation from December to February 2014, the fungal colonization pattern remained consistent throughout these periods (Fig. 3B and C). These results suggest that the annual new colonization correlates more strongly with RH than with rainfall. Linear regression analysis supported this observation, revealing a comparatively strong correlation between monthly *Lasiodiplodia* colonization incidence and relative humidity (*R*² = 0.70; Fig. S6A), while a weaker correlation was observed with monthly rainfall (*R*² = 0.25; Fig. S6B).

### Higher humidity favors pycnidiospore release

**Fig 4:**
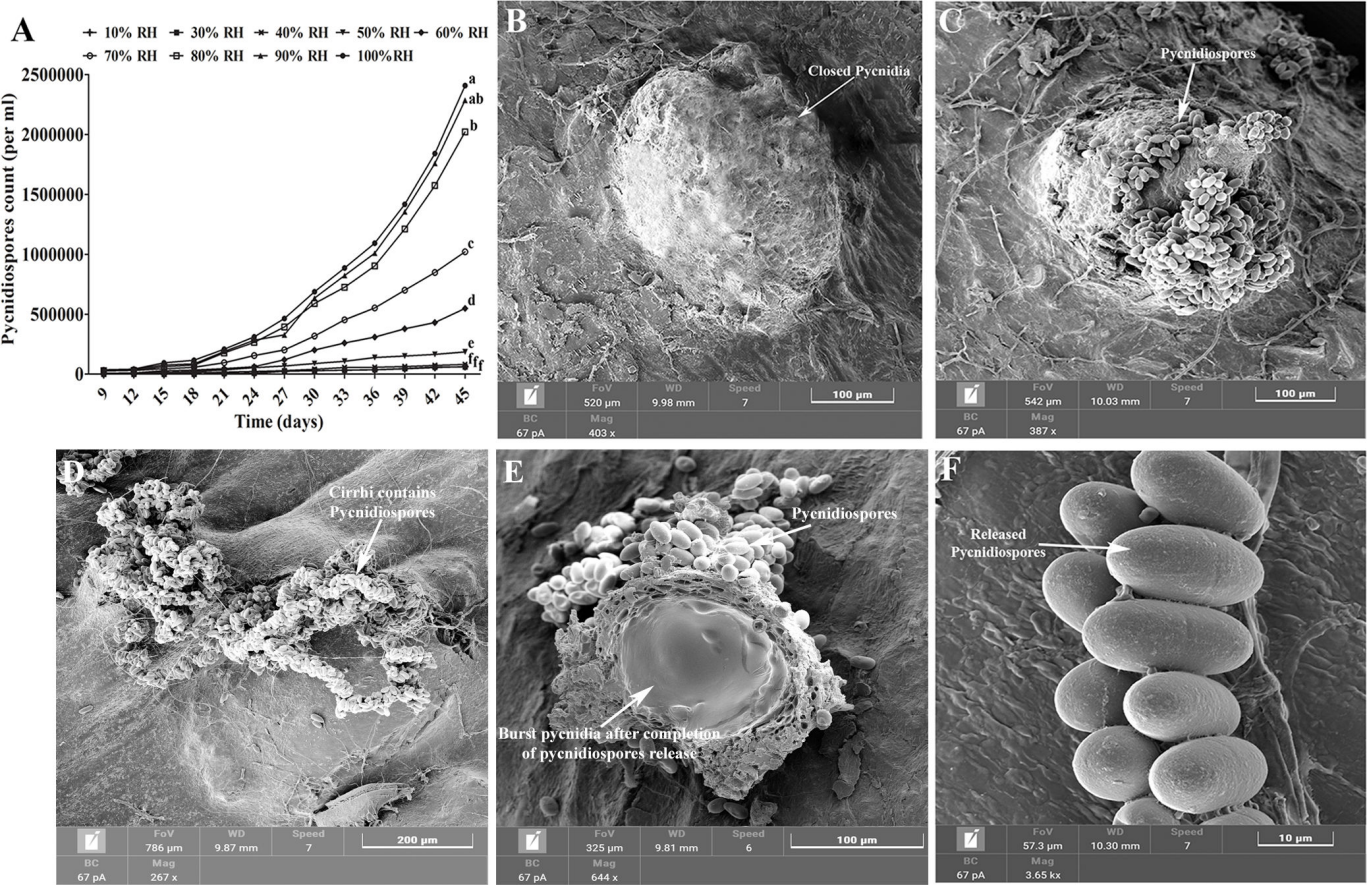
Impact of RH on pycnidial burst and pycnidiospore release. (**A**) Young, green avocado twigs were inoculated with *L. theobromae*. The twigs were incubated in chambers with different RH, and the released pycnidiospores were recorded at different time points. Values are mean ± standard error (SE). Different letters indicate significant differences according to Tukey’s multiple comparison test (*P* ≤ 0.05); (**B**) closed pycnidia at 10% RH; (**C**) initiation of pycnidial burst at 100% RH; (**D**) cirrhus-like structures localized on the pycnidial burst site at 100% RH; (**E**) pycnidial structure after a complete burst; (**F**) released pycnidiospores after burst.

Different levels of RH were tested *in vitro* to evaluate their impact on the pycnidiospores released from pycnidia formed on infected avocado twigs. The results demonstrated that as RH levels increased, there was a corresponding rise in the number of pycnidiospores released. A significant impact was found between the different RH levels after 18 days of infection. At 45 days of incubation under various humidity levels, the release of pycnidiospores was 38.2-fold (*P* < 0.0001) higher at 100% RH compared to 10% RH, followed by 90% RH (36.2-fold; *P* < 0.0001), 80% RH (32.1-fold; *P* < 0.0001), and 70% RH (16.2-fold; *P* < 0.0001) (Fig. 4A). Moreover, the maximum pycnidiospores released were recorded at 100% RH (2.4 × 10^6^), which was relatively close to 90% and 80% RH (2.02–2.28 × 10^6^), respectively (Fig. 4A). No significant differences in the release of pycnidiospores were found between 10%, 30%, and 40% RH. SEM was used to further investigate the impact of RH on the release of pycnidiospores from infected twigs. The closed pycnidia (at 10% RH) were seen as a hill on the twig surface without releasing pycnidiospores (Fig. 4B). However, at 100% RH, the surface of the infected twigs was characterized by numerous pycnidial bursts, which resulted in pycnidiospore release (Fig. 4C through F). These observations further demonstrate that high RH promotes the bursting of pycnidia and the subsequent release of pycnidiospores, whereas low RH conditions result in unopened pycnidia with no spore release.

### Diurnal aerial dispersal of pycnidiospores in rainless days

**Fig 5:**
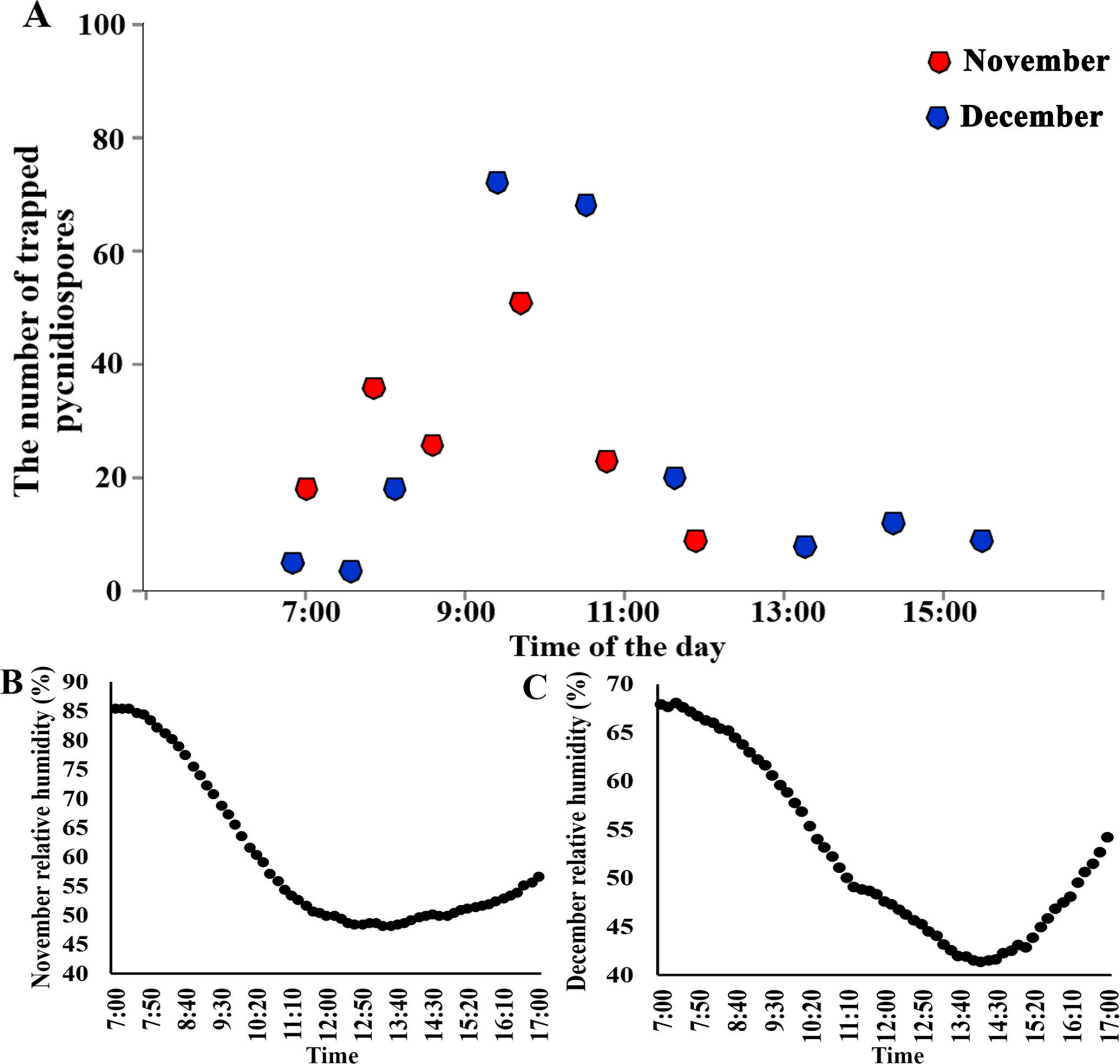
Diurnal aerial dispersal. (**A**) Aerial dispersal of pycnidiospores in an avocado orchard on rainless days of November and December. (**B and C**) Relative humidity (%) of the orchard in respective months.

To characterize the diurnal aerial dispersal of pycnidiospores, spore traps were placed in a commercial avocado orchard on rainless 2 representative days of November and December. Moreover, patterns of the aerial dispersal of pycnidiospores were comparable on both sampling dates. In the early morning, when the morning dew started to dry, and the RH was high, ~85% in November and ~67% in December (Fig. 5B and C), the dispersal rate was very low. As the day progressed, RH decreased gradually, and the highest spore dispersal rate was recorded at about 10:00 a.m., when RH was below 55%. Afterward, the trapping of pycnidiospores decreased and remained low until the end of the measurement (Fig. 5A). The findings indicated that the diurnal dispersal of pycnidiospores increased with sunrise and decreased in RH. Notably, no pycnidiospores were captured on rainless summer days characterized by low diurnal humidity.

### Factors affecting the aerial dissemination of pycnidiospores

**Fig 6:**
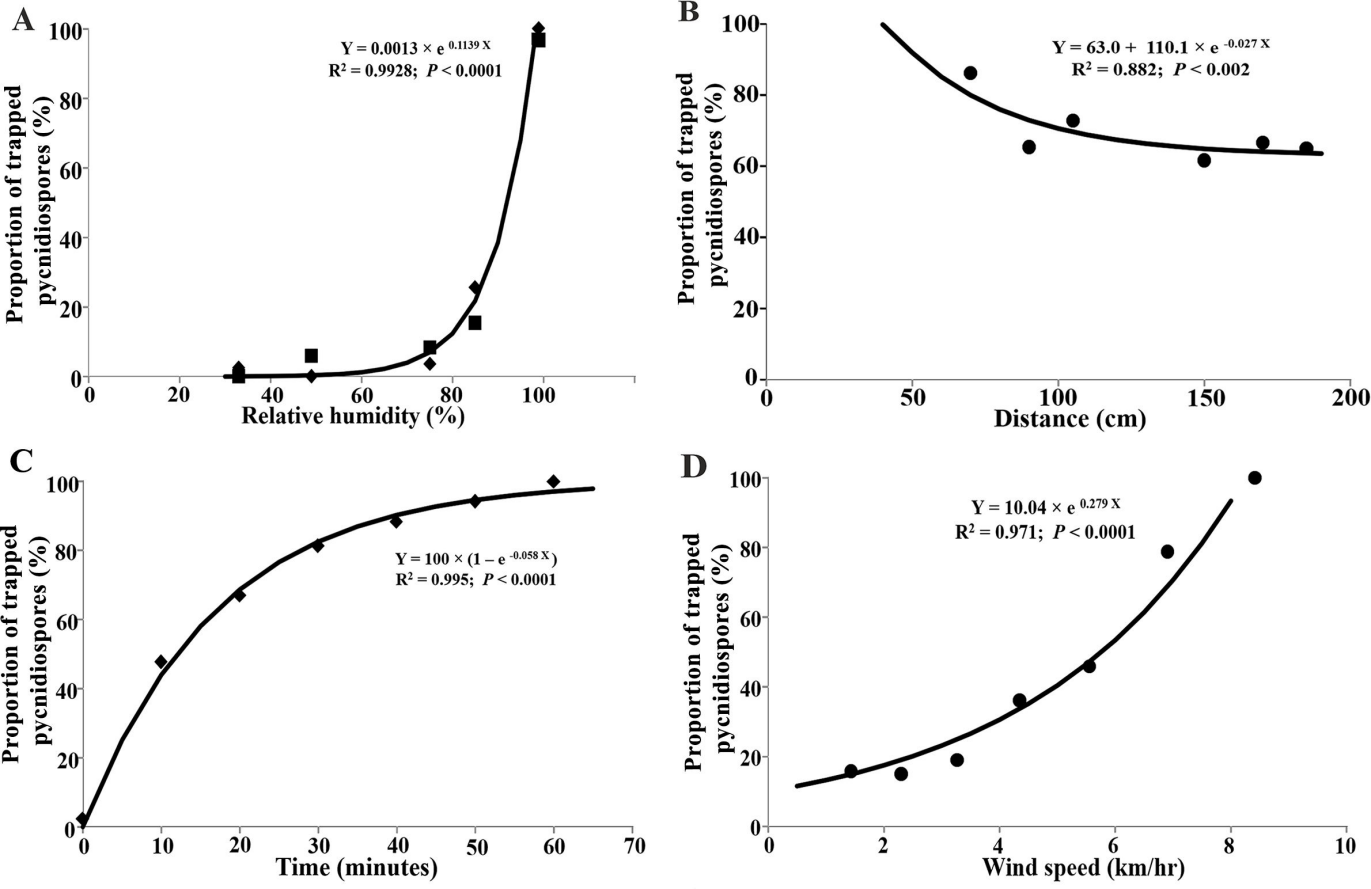
Factors affecting the aerial dissemination of *L. theobromae* pycnidiospores in a wind tunnel. (**A**) Impact of relative humidity on the release and spread of pycnidiospores from infected twigs; (**B**) the distance at which airborne pycnidiospores were dispersed in the wind. (**C**) The time rate at which released pycnidiospores were dispersed by wind; (**D**) **e**ffect of wind speed on the aerial dispersal of released pycnidiospores.

Air-tunnel-based *in vivo* experiments were conducted to study the factors affecting the aerial release of pycnidiospores. The observations further revealed that high RH correlates with *L. theobromae* pycnidiospore release via pycnidial burst (Fig. 4A and 3F). The relationship between RH level and pycnidiospore release was exponential; below 70% RH, the relative number of released pycnidiospores was minimal, which exponentially increased with increased RH (Fig. 6A).

The incidence of trapped pycnidiospores decreased with the tunnel length, while a significant proportion of pycnidiospores (70%) were trapped at the tunnel end (Fig. 6B). This indicates that once the pycnidiospores were released and became airborne, the distance of their dispersal was larger than the length of the air tunnel.

When exposed to a constant wind of 2.22 m s^−1^, about 50% of the released pycnidiospores were rapidly dispersed within the first 10 min. Thereafter, the proportion of dispersed pycnidiospores decreased gradually, and after 60 min, the release of pycnidiospores was complete (Fig. 6C). Similarly, wind speed affected the release of pycnidiospores, and the proportion of released pycnidiospores increased as wind speed intensified (Fig. 6D). Twenty percent of the released pycnidiospores were dispersed even in light air current (0.56 m s^−1^), while at light breeze (2.5 m s^−1^), all pycnidiospores were dispersed (Beaufort wind scale).

### Biochemical mechanism governing pycnidial burst

#### Osmolytes assessment in pycnidial sap

**Fig 7:**
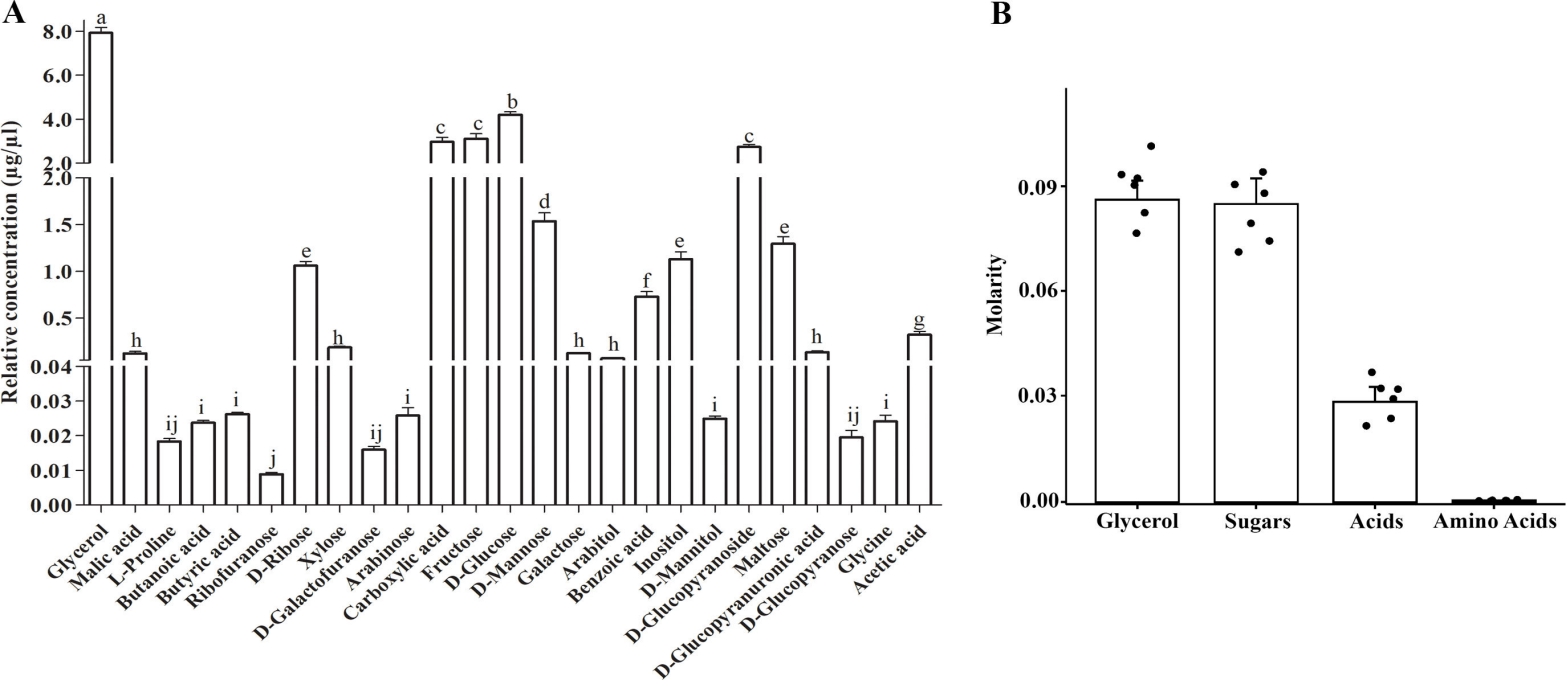
GC-MS profiling of pycnidial sap. (**A**) The relative concentration of different compounds in the closed pycnidial sap. Values are mean ± SE. Different letters indicate significant differences according to Tukey’s multiple comparison test (*P* ≤ 0.05). (**B**) Groups of compounds found in pycnidial sap, as well as small black circles, represent individual values.

Different concentrations of osmolytes were assessed from sap of an average of 1,151.8 closed pycnidia per experiment in biological triplicate, over six independent experiments. The gas chromatography mass spectrometry (GC-MS) profiling of the derivative compounds from the closed pycnidia confers the predominance of 25 major compounds in the pycnidial sap (Fig. 7A), which could be classified into four groups (Fig. 7B). Out of those compounds, we found that the group of sugars (15.67 ± 0.66 µg µL^−1^) and glycerol (7.93 ± 0.50 µg µL^−1^) were the main components that altered the pycnidial sap molarity, along with acids (4.19 ± 0.11 µg µL^−1^) and amino acids (0.04 ± 0.005 µg µL^−1^) (Fig. 7B). Within the group of sugars, we identified glucose (4.2 ± 0.26 µg µL^−1^), fructose (3.11 ± 0.45 µg µL^−1^), glucopyranoside (2.73 ± 0.24 µg µL^−1^), mannose (1.53 ± 0.18 µg µL^−1^), maltose (1.29 ± 0.15 µg µL^−1^), and ribose (1.06 ± 0.09 µg µL^−1^). In the group of acids, we found carboxylic acid (2.97 ± 0.30 µg µL^−1^), benzoic acid (0.73 ± 0.011 µg µL^−1^), and acetic acid (0.32 ± 0.02 µg µL^−1^) (Fig. 7A). The molarity of the categorized groups of glycerol, sugars, acids, and amino acids was 0.086 M, 0.085 M, 0.028 M, and 0.0005 M, respectively (Fig. 7B). Thus, GC-MS profiling revealed that the major compounds present in the closed pycnidial sap are sugars and glycerol, which lead to a relatively high cumulative molarity of 0.171 M, and together with acids and amino acids, the molarity reached 0.2 M.

#### Evaluation of atmospheric pressure in closed pycnidia

The GC-MS profiling of the relative concentration of compounds in the closed pycnidial sap was converted into molar concentration to assess the atmospheric pressure of closed pycnidia before the pycnidial burst. The cumulative molar concentration of pycnidial sap osmolytes, that is, sugars and glycerol, resulted in a pressure of approximately 4.18 ATM. This finding indicates that the pressure inside the pycnidia was higher than the external environmental atmospheric pressure, which is close to one atmosphere. Interestingly, after reaching balance with the environment, the cumulative concentration of compounds in the already erupted pycnidia reached 0.041 M, which led to a pressure of one atmosphere (Fig. S7A and B). Thus, the accumulation of sugars and glycerol increases the internal pressure within the pycnidia, which likely facilitates the rupture of the pycnidia and the subsequent release of pycnidiospores.

## DISCUSSION

Fungal phytopathogens account for a major proportion of plant diseases globally, with estimates attributing 70%–80% of all plant pathogenic infections to fungi and posing a significant threat to food security (36, 37). These pathogens are sessile organisms, incapable of independent movement. As a result, large-scale dispersal and the establishment of new infections heavily rely on spore dispersal, often driven by environmental factors. To facilitate this process, many fungal pathogens have evolved specialized mechanisms for spore release and dissemination (38–40). Pycnidia are specialized asexual fruiting bodies that disperse pycnidiospores over short distances by water splash and are produced by several Ascomycota lineages, including the orders Sphaeropsidales or Pleosporales (19).

Within the Ascomycota phylum, fungi from the *Botryosphaeriaceae* family are widespread pathogenic fungi known to produce abundant asexual pycnidia (18). The *Botryosphaeriaceae* family comprises around 200 species belonging to the Ascomycota phylum (33, 41). Most of these species are pathogens and can infect over 650 host plants globally (22, 34, 35, 42). They typically colonize host plants through injuries and natural openings (43, 44), persist endophytically in vascular tissues without showing symptoms until environmental stress or fruit maturation occurs. Thereafter, they switch to a necrotrophic lifestyle, causing symptoms such as dieback and stem-end rot (45).

Like other Ascomycota, *Botryosphaeriaceae* can reproduce through wind-dispersed sexual ascospores (18, 46, 47). However, this sexual stage was rarely observed worldwide (29, 48–50) and has never been documented in Israel (28). Consequently, asexual pycnidiospores are released from pycnidia and spread through water splashes (13, 19, 21, 30, 51). Nevertheless, several studies have reported the presence of pycnidiospores in the air and their dispersal during rainless periods, suggesting that aerial transport of asexual spores can occur under suitable environmental conditions such as relative humidity and wind (21, 28, 31). Similarly, many disease epidemics were associated with asexual spores spread by water splash (8, 14, 40, 52). However, the mechanistic basis linking environmental factors, particularly relative humidity dynamics, to pycnidial rupture and subsequent aerial dispersal has remained unresolved. Numerous observations from commercial avocado plantations (Fig. 1) and other orchards and forests indicate that *Botryosphaeriaceae* dieback symptoms have spread aerially across considerable distances, consistent with aerial dissemination. A closer evaluation revealed that this pattern was correlated with pycnidial burst and release of pycnidiospores (Fig. 1), suggesting a potential role for asexual spores in disease spread under field conditions.

To evaluate whether this phenomenon is global for a wide host range of phytopathogens belonging to the *Botryosphaeriaceae* family, we inoculated five genera, that is, *Lasiodiplodia, Neoscytalidium, Neofusicoccum, Diplodia*, and *Dithiorella,* of fungal pathogens on different twigs from various crops, including evergreen species from subtropical origin such as mango, Mediterranean origin as olive, deciduous fruits like nectarine, table grape, and citrus varieties such as mandarin. Additionally, we inoculated twigs from natural ecosystems, including oak and pine trees. The tested *Botryosphaeriaceae* species induced pycnidial burst and pycnidiospores release on all tested twigs from different trees (Fig. 2 (1a–5g)). SEMs of different infected twigs revealed that while the general mechanism of asexual spore release (driven by pycnidia burst) was conserved across all tested pathogens, variations in the specific release patterns were evident. These variations in pycnidiospore release patterns may be attributed to the structural diversity of pycnidia between different *Botryosphaeriaceae* genera (18, 32, 53). Interestingly, the minimal impact of host plant on pycnidia burst structure and spore release indicates that these mechanisms are primarily pathogen-driven and less influenced by host factors. This consistency across hosts simplifies the predictions of *Botryosphaeriaceae* infections in diverse agroecosystems.

While pycnidia are abundant in various orchards, the factors influencing pycnidiospore release and their long-distance dispersal have remained poorly understood. *L. theobromae* on avocado trees was used as a model to better understand the mode of action of the pycnidial burst. The new colonization pattern of young avocado twigs recorded over 3 consecutive years suggests that the inoculum dispersal was, in most instances, not related to rainfall events. The increase in colonization rate of young green avocado twigs was initiated in mid-summer (July–August), several months before the beginning of the rainy season (Fig. 3). While part of the observed colonization may reflect the expansion of pre-existing latent endophytic populations, the consistent temporal pattern in young green twigs was associated with environmental conditions, which supports the hypothesis that the spores of *Botryosphaeriaceae* can disperse aerially in rainless conditions. Additionally, the RH showed a much stronger correlation with the establishment of *Lasiodiplodia* disease symptoms (Fig. 3B; Fig. S1). The linear regression analysis also indicated that *Lasiodiplodia* new colonization incidence is better correlated to relative humidity than rainfall, suggesting that the new colonization is not dependent only on rainfall events but mainly on the RH patterns during rainless days (Fig. S6A and B). This aligns with findings that species of *Botryosphaeriaceae*, like *Lasiodiplodia*, require moist environments for proliferation (13, 54–57). Indeed, an elevation in the incidence of infection commonly occurs under high RH in different hosts like grapevine (21), peach (58), mango (59), and loquat (60). To better understand the RH effect on pycnidial burst, *L. theobromae*-infected twigs were incubated at various levels of RH, which showed a significant increase in pycnidial release and dispersal above 70% RH (Fig. 4 and 6). The results from both experiments emphasize RH’s pivotal role in releasing pycnidiospores. Further investigations also suggested that RH has a role in the release of pycnidiospores; however, it was based on assumptions or observations of spore count in the field (13, 17, 21, 29, 61, 62). Interestingly, SEMs taken under different RH conditions showed that under high RH, the pycnidia burst, while at low RH, the pycnidia formed but did not burst (Fig. 4). A deeper investigation found that on rainless days, the highest aerial dispersal was recorded during dry conditions after sunrise (Fig. 5A). This correlated well with the decrease in RH along the day (Fig. 5B and C). This finding suggests that high RH likely triggered the release of pycnidiospores, followed by sunrise and reduced RH later in the day. This can dry the mucilage around the released pycnidiospores and trigger their aerial dispersal in the orchard. This result agrees with a study showing that aerial dispersal of *Lasiodiplodia* sexual spores was intensified between early morning and 10:00 a.m. in coconut plantations (63). This phenomenon is similar to airborne urediniospores of *Phakopsora pachyrhizi* from soybean (64, 65) and uredospores of *Hemileia vastatrix* from coffee (66), which showed a positive correlation between the high aerial release of spores and the decrease in RH and increase in wind speed.

The dispersal of pycnidiospores increased significantly with exposure time (Fig. 6C) and wind speed (Fig. 6D) in a wind tunnel experiment, suggesting that pycnidiospore emission is strongly wind driven and may facilitate their potential transport under favorable environmental conditions. The dispersed pycnidiospores traveled more than 2 m (Fig. 6B), the length of the wind tunnel, and could probably be carried by the wind for much larger distances. Similarly, other studies showed that intense turbulent winds could carry airborne spores over long distances (5, 67).

To address the knowledge gap in the mechanism of pycnidia burst and pycnidiospore release, we investigated the composition of osmolytes in pycnidial fluid before the pycnidia burst. Our analysis revealed high concentrations of sugars and glycerol accumulating within the closed pycnidia (Fig. 7). Soluble sugars are widely recognized as osmotically active solutes (68, 69). Sugars and glycerol are predominantly found in the appressoria of *Colletotrichum higginsianum* and *Phakopsora pachyrhizi*, which create osmotic pressure that reaches ~50 ATM (70–72). Glycerol accumulation generates enormous turgor pressure in the appressorium of *Magnaporthe oryzae* (73, 74) and *Metarhizium robertsii* (75). Using this force, the fungi release a peg that penetrates the plant cuticle and cell wall. Moreover, glycerol is a hyperosmotic agent and results from the breakdown of lipids and glycogen (71, 76). Similarly, the high concentrations of glycerol and sugars possibly perform a decisive role necessary for the pycnidial burst as they increase osmotic pressure inside the pycnidia when dissolved in water that penetrates and infuses into closed pycnidia at high RH conditions. The concentration of sugars and glycerol resulted in a high molarity of 0.171 M in the pycnidial sap, leading to ~4.18 ATM pressure inside the closed pycnidia. These results suggest that a buildup of pressure inside fungal pycnidia, higher than the surrounding atmospheric pressure, could lead to the pycnidia bursting. The pressure in a pycnidium that needs to be opened should be higher than 1 ATM, but could be lower than the pressure in the appressoria that needs to create a powerful enough force to drill into the fruit cuticle and cell wall. These results are aligned with previous studies on the biomechanics of ascospores opening, where high sugar concentration in the ascus sap of *Gibberella zeae* initiated engorgement and turgor pressure, leading to the initial expansion of the ascus wall (10, 77). Glycerol was also recognized as one of the major constituents in the epiplasmic fluid of the apothecium of the fungus *Ascobolus immerses*. It accounted for 0.98 ATM out of 3.06 ATM pressure in the ascus sap, while the maximum turgor pressure generated inside the ascus was 4.44 ATM. However, 1.97 ATM was sufficient to force the cluster burst of ascospores over a distance (78). This is consistent with our results, where only glycerol and sugars individually created 2.1 and 2.0 ATM pressure (together 4.1 ATM) in the closed pycnidia for burst.

**Fig 8:**
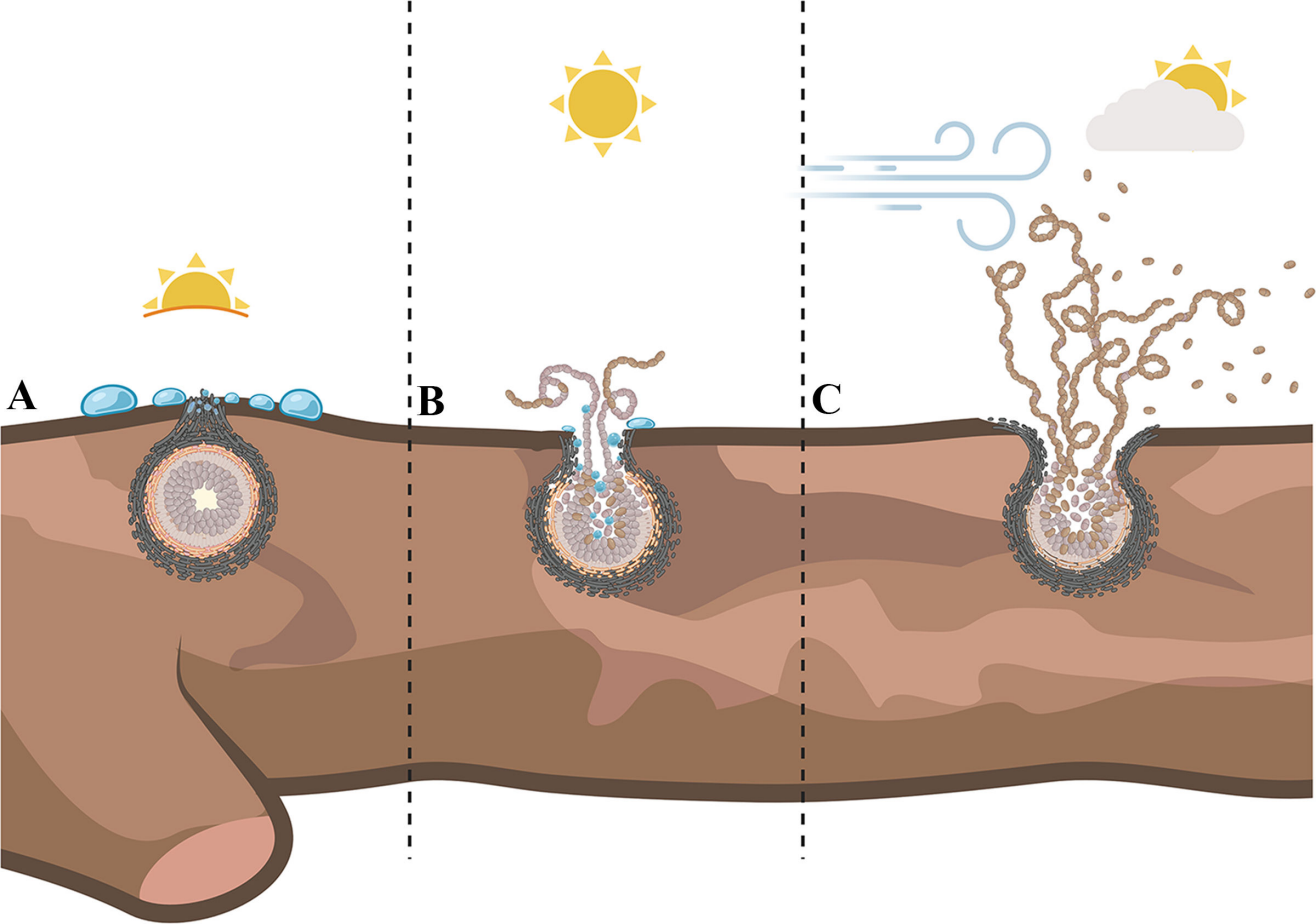
Proposed pycnidiospore release mechanism along the day. (**A**) In the early morning, high RH allows moisture to penetrate the thin wall of closed pycnidia; (**B**) the moisture mixed with pycnidial sap increases osmotic pressure and triggers the pycnidial burst at ~4.18 ATM, and then the pycnidial sap slowly dries throughout the day; (**C**) the released pycnidiospores dry and disperse by the wind.

Based on our findings, we propose the potential mechanism governing the stages leading to pycnidial burst and long-distance dispersal of pycnidiospores: in the early-winter mornings, elevated RH and morning dew condensation lead to water permeating into the outer wall of the closed pycnidia (Fig. 8A). The influx of water mixed with the pycnidial sap that contains a high concentration of osmolytes, that is, glycerol and sugars (Fig. 7A and B), elevates the osmotic pressure, ultimately triggering the pycnidial burst (Fig. 8B). Subsequently, pycnidiospores with pycnidial sap are released from the pycnidia, appearing like tiny droplets on the infected twig. As the morning progresses, the RH decreases, the temperature rises, and the sunlight intensity starts to dry the mucilage around the released pycnidiospores. Consequently, the dried pycnidiospores disperse and are carried over large distances by the wind (Fig. 8C).

This study provides the first mechanistic evidence that high RH induces osmotic pressure within the closed pycnidia, which leads to the release of pycnidiospores through pycnidial burst, which later is carried by the wind. This model of spreading mechanism may also apply to other fungi belonging to the *Botryosphaeriaceae* and *Diaporthaceae* families, such as *Septoria, Ascochyta, Diplodia, Phoma*, and others, suggesting a broader ecological relevance. The proposed mode of action of pycnidial bursts provides new insights for the development and adaptation of disease management strategies for disease outbreaks in agroecological settings. By better understanding of environmental and meteorological factors influencing pycnidial burst and aerial dispersal of pycnidiospores, we can predict and mitigate disease spread in both agricultural and forest ecosystems under variable environmental conditions.

## MATERIALS AND METHODS

### Assessment of pycnidial burst from several plantations infected by *Botryosphaeriaceae* pathogens

Healthy, young green twigs (10 cm–12 cm) of different agricultural and forest plants, including Mango (*Mangifera indica*), Olive (*Olea europaea*), Nectarine (*Prunus persica*), Table grape (*Vitis vinifera*), Mandarin (*Citrus reticulata*), Oak (*Quercus* spp.), and Pine (*Pinus* spp.), were collected and surface sterilized. To eliminate resident endophytic and latent microbial contaminants, twigs were autoclaved prior to inoculation, ensuring that fungal colonization and sporulation resulted exclusively from the inoculated *Botryosphaeriaceae* species. The sterilized twigs were placed on soft agar medium in autoclaved containers and infected individually with *Lasiodiplodia theobromae* (B-43)*, Neoscytalidium dimidiatum* (B-47)*, Neofusicoccum parvum* (B-33)*, Diplodia neojuniperi* (B-37)*,* or *Dethiorellla viticola* (B-38). For each host-pathogen combination, 12 independent twigs of each plant species were used (*n* = 12 biological replicates). The infected twigs were incubated at 24°C under high humidity (70%–80% RH) for 3–4 weeks to examine the development of pycnidial structures. The entire experiment was repeated four times independently under identical conditions to ensure the reproducibility. To assess the pattern of pycnidiospore release upon pycnidial burst, three representative twigs of each plant species from each pathogen were randomly selected from each experimental set for microscopic observation. The pycnidial morphology and pycnidiospore release pattern of individual pathogens on different plant species were visualized under a high-resolution scanning electron microscope (Tescan Mira 4 FEG SEM, USA).

### Seasonal colonization patterns of *Lasiodiplodia*

Colonization by members of *Botryosphaeriaceae* in young, green avocado twigs was monitored from 2014 to 2016 in “Hass” avocado orchard (31°27′36″ N, 34°37′22″ E). Every 1–2 months, 33–35 healthy green twigs (10 cm–15 cm long) were randomly sampled from different trees. The sampled twigs were surface sterilized by NaOCl 1% for 1 min, plated on potato dextrose agar (PDA), and incubated at 22°C. Emerging spores from fungal colonies were initially examined using light microscopy to identify typical *Botryosphaeriaceae* morphotypes. Representative isolates with distinct spore morphology were identified by combined morphological and molecular approach. The genomic DNA of each isolate was extracted and the internal transcribed spacer (ITS) region was amplified by PCR using universal primers ITS1 and ITS4 under standard conditions (e.g., initial denaturation at 95°C for 5 min; 35 cycles of 95°C for 30 s, 55°C for 30 s, 72°C for 1 min; and final extension at 72°C for 10 min). Amplicons of ITS region were purified, sequenced (by Sanger sequencing), and compared to the GenBank references using BLAST for species-level identification. Morphological characteristics, including colony and conidial features, were also used to support species identification. Using this combined approach, isolates were identified at species level. Incidence (%) of colonization was calculated, and a sinus regression equation model was applied to 3 year data. Monthly mean RH values and cumulative monthly rainfall data were obtained from a weather station operated by the Israel Meteorological Service (IMS; https://ims.gov.il/en) located 2 km from the orchard. Linear regression analysis was performed to examine the relationship between the monthly new colonization incidence of *Lasiodiplodia* and relative humidity or rainfall at the respective time.

### Effect of RH in *in vitro* conditions

Surface-sterilized and autoclaved avocado twigs (10 cm–12 cm) were infected by placing an agar plug (6 mm in diameter) containing *L. theobromae* mycelium at the cut end of each twig. The fungus was isolated from an infected avocado tree in Israel (24). Infected twigs were incubated at 25°C in different sealed humidity chambers with different RH using specific saturated salt solutions, as follows: LiCl (10.95%–11.35 % RH), CH_3_CO_2_K (22%–23%), MgCl_2_ (29%–35%), Mg(NO_3_)_2_ (45%–60%), NaNO_2_ (61%–65%), NaCl (74%–76%), KCl (80%–89%), KNO_3_ (85%–96%), and distilled water (100%) (79). These saturated salt solutions were used to establish and maintain defined and stable RH levels within closed chambers. The inoculated twigs were placed inside the humidity chambers and sealed with cling film (10 µm). The RH level in the different chambers was regularly monitored using a portable thermohygrometer (Rotronic Hygrolog HL-1D, Bassersdorf, Switzerland). The inoculated twigs were sampled on the 40th day to observe the formation of pycnidial structures after *L. theobromae* inoculation for other observations (Fig. S1). The effect of RH on the release of pycnidiospores from developed pycnidia was quantified every 3 days over a 45 day period, and results were expressed as the number of pycnidiospores per twig per sampling time. Each treatment of RH level consisted of 21 replicates (*n* = 21), and the experiment was conducted twice independently.

### SEM

SEM analysis was performed to validate the impact of RH on the release of pycnidiospores from the *L. theobromae* pycnidia in avocado plants. Infected twigs that contained pycnidia (*n* = 5) were incubated at 10% or 100% RH conditions, were randomly excised under a stereo microscope, and longitudinally sectioned into small pieces (0.2 cm–0.4 cm). The sections of infected twigs were further processed for SEM imaging (80). The dehydrated sections of respective treatments were further coated with gold-palladium in a sputter coater (SC7620 Mini Sputter Coater, Quorum, UK). The coated sections, comprising three independent samples of each treatment, were visualized using high-resolution field emission SEM (TESCAN MIRA 4 FEG SEM, USA).

### Diurnal aerial dispersal of pycnidiospores

The diurnal aerial dissemination of *Lasiodiplodia* pycnidiospores was recorded in the same avocado orchard using a volumetric air sampler (WD31PJ, Burkard Manufacturing, Ltd., Hertfordshire, UK) (Fig. S2). The sampler draws air at a constant flow rate and directs airborne spores onto adhesive-coated glass microscope slides. Each trap accommodated five to six adhesive-coated (e.g., petroleum jelly) microscope slides at once for sequential spore collection. Five spore traps were installed between the rows of trees on two represented rainless days of November and December, and on six rainless summer days with low diurnal humidity. Sampling commenced at 06:40 a.m., with traps activated at 30 min intervals for 10 min per sampling event. After each interval, the microscope slides were carefully removed and replaced with freshly coated slides for the next sampling event. Collected slides were subsequently visualized under a light microscope (×100 magnification) to quantify *Lasiodiplodia* pycnidiospores and expressed as the number of pycnidiospores trapped per sampling interval. RH data corresponding to each sampling time were obtained from the IMS.

### Factors affecting the aerial dissemination of pycnidiospores using a wind tunnel

Green twigs (10 cm–12 cm) of “Hass” avocado were excised; autoclaved, and five twigs were fixed in 10 cm pots filled with perlite over a 5 cm water layer. A 6 mm agar block of *L. theobromae* was placed on the tip of each twig. The inoculated twigs were incubated at 23 ± 2°C under high RH for 2 weeks to allow pycnidial development. Subsequently, stem segments were transferred to closed containers filled with saturated salt solutions (2 cm–5 cm depth) to establish different RH levels, as follows: MgCl (33%); KNO_2_ (49%); NaCl (78%); KCl (85%); distilled water (100%), which were verified with data loggers (Hobo; U23-001). After 48 h of incubation in RH containers, the number of pycnidiospores released from the pycnidia was quantified using a wind tunnel equipped with a peripheral air blower (Degania Sprayers, Degania, Israel) with an outlet opening of 330 cm^2^ (Fig. S3A through C).

Infected twigs exposed to the treatments specified above were placed in front of the air blower and served as the inoculum source. Wind speed near the infected twigs was measured using a Pitot tube anemometer (SwcmaMann, Farsta, Sweden) and maintained at to 2.22 m s^−1^. Spore traps were positioned within the wind tunnel, and pycnidiospores were collected during 1 h of exposure of infected twigs under continuous airflow, with microscopic slides that were replaced every 10 min. Additional experiments assessed the effects of wind speed (0.28–2.22 m s^−1^) and distance on the dissemination of pycnidiospores, where spore traps were placed 70 cm–200 cm from the inoculum source (Fig. S3).

### GC-MS profiling of pycnidial sap

The chemical composition of pycnidial sap was analyzed using GC-MS. Mature, closed pycnidia (16–18 days after inoculation at 100% RH) were sampled from infected twigs (Fig. S4), with an average of 1,151.8 closed pycnidia sampled across six independent experimental repetitions. Pycnidial sap was released by inducing pycnidial burst in 1 mL of double-distilled water, confirmed by light microscopy (Fig. S5). The resulting mixture containing ruptured pycnidia underwent centrifugation at 10,000 rpm for 5 min, and the supernatant containing pycnidial fluid was extracted and placed into a sterile reaction tube. Sap from naturally erupted pycnidia was similarly collected and processed in the same manner for comparative analysis. The supernatant was lyophilized (until 100 µL remains) and stored at 4°C prior to derivatization. The collected sample of pycnidial sap (100 µL) was derivatized (81) using ribitol (Sigma-Aldrich) as an internal standard and stored at −20°C in a 2 mL autosampler vial. Briefly, the derivatized sample (1 µL) of pycnidial sap was introduced by an autoinjector into the inlet of an Agilent 7890A gas chromatograph equipped with an Agilent HP-5MS column (30 m × 0.25 mm × 0.25 μm) and coupled with the MS detector (Agilent 5977B). Helium (99.9% purity) served as the carrier gas under constant pressure mode with a flow rate of 1 mL min^−1^. The GC-MS run of 22.67 min included a program: column temperature of 40°C was maintained for 2 min, followed by an increase of 10°C per minute until reaching 150°C, and held for 10 min at 150°C. The temperature was then increased to 220°C at a rate of 15°C per minute and held at 220°C for 5 min. The ionization energy of the mass spectrometer was adjusted to 70 eV for the analysis. The compounds in pycnidial sap were identified using the NIST mass spectral library, Version 5 (Agilent), and MSD Chemstation E.02.00.493 based on retention indices and mass spectra. The peak area of each identified compound was quantified for relative concentration (µg µL^−1^) and compared to the internal standard concentration.

### Assessment of pressure inside the pycnidia

Sap was taken from the closed pycnidia sampled from infected twigs incubated under high relative humidity (>80%) to ensure hydration and structural integrity. Similarly, sap under the same conditions was collected separately after adding ddH_2_O to open pycnidia for comparative analysis. The osmolyte composition of pycnidial sap was analyzed using GC-MS, and concentrations of individual compounds were expressed in molarity. Thereafter, the cumulative molarity value of all the compounds was used to calculate the atmospheric pressure in the pycnidia using the formula:


$$P=\frac{nRT}{V}$$

where *P* is pressure (ATM), *V* is volume (liter), *n* is number of moles of total compound, *R* is 0.821 L·ATM mol^−1^ K^−1^ (ideal constant), and *T* is temperature (Kelvin).

This approach provides an estimate of osmotic pressure within closed pycnidia. Comparative analysis of osmolyte composition in closed and open pycnidia was used to evaluate differences in internal pressure associated with pycnidial burst and pycnidiospore release.

### Statistical analysis

The experimental observations were statistically analyzed using SPSS version 18.0 software (SPSS Japan, Tokyo, Japan). Tukey’s multiple-comparison test (*P*< 0.05) was employed to calculate the significance level between means. Non-linear regression analyses were used to analyze the results of wind tunnel experiments. In each experiment, the number of pycnidiospores trapped in the treatment with the highest value was defined as 100%, and the other treatments’ records were adjusted proportionally. The dependent variable (*y*-axis) was the proportion of trapped pycnidiospores (%). The independent variables (*x*-axis) were RH (%), time with high RH (in hours), temperature (in °C), wind speed (in km/h), and dispersal distance (in cm). Data from the repeated experiments were pooled before analysis. The specific regression equation was chosen based on the *P*-value, coefficient of determination (*R*^2^), and MSE. Moreover, GraphPad Prism version 8 (GraphPad Software, Boston, MA) was used for the linear regression analysis to assess the correlation between monthly *Lasiodiplodia* new colonization incidence and corresponding relative humidity and rainfall values.

## Data Availability

The data set assembled for this study is available in the supplemental material.

## SUPPLEMENTAL MATERIAL

Supplemental figures msphere.00034-26-s0001.docx Fig. S1 to S7.

ASM does not own the copyrights to Supplemental Material that may be linked to, or accessed through, an article. The authors have granted ASM a non-exclusive, world-wide license to publish the Supplemental Material files. Please contact the corresponding author directly for reuse.

## References

[1] Ristaino JB, Anderson PK, Bebber DP, Brauman KA, Cunniffe NJ, Fedoroff NV, Finegold C, Garrett KA, Gilligan CA, Jones CM, Martin MD, MacDonald GK, Neenan P, Records A, Schmale DG, Tateosian L, Wei Q. 2021. The persistent threat of emerging plant disease pandemics to global food security. Proc Natl Acad Sci USA 118:e2022239118. doi:10.1073/pnas.2022239118 34021073 PMC8201941

[2] Fones HN, Bebber DP, Chaloner TM, Kay WT, Steinberg G, Gurr SJ. 2020. Threats to global food security from emerging fungal and oomycete crop pathogens. Nat Food 1:332–342. doi:10.1038/s43016-020-0075-0 37128085

[3] Fisher MC, Gurr SJ, Cuomo CA, Blehert DS, Jin H, Stukenbrock EH, Stajich JE, Kahmann R, Boone C, Denning DW, Gow NAR, Klein BS, Kronstad JW, Sheppard DC, Taylor JW, Wright GD, Heitman J, Casadevall A, Cowen LE. 2020. Threats posed by the fungal kingdom to humans, wildlife, and agriculture. mBio 11:e00449-20. doi:10.1128/mBio.00449-20 32371596 PMC7403777

[4] Chaudhary VB, Aguilar-Trigueros CA, Mansour I, Rillig MC. 2022. Fungal dispersal across spatial scales. annual review of ecology, evolution, and systematics. Annu Rev Ecol Evol Syst 53:69–85. doi:10.1146/annurev-ecolsys-012622-021604

[5] Lagomarsino Oneto D, Golan J, Mazzino A, Pringle A, Seminara A. 2020. Timing of fungal spore release dictates survival during atmospheric transport. Proc Natl Acad Sci USA 117:5134–5143. doi:10.1073/pnas.1913752117 32098849 PMC7071907

[6] Roper M, Seminara A, Bandi MM, Cobb A, Dillard HR, Pringle A. 2010. Dispersal of fungal spores on a cooperatively generated wind. Proc Natl Acad Sci USA 107:17474–17479. doi:10.1073/pnas.1003577107 20880834 PMC2955148

[7] Halbwachs H, Bässler C. 2015. Gone with the wind – a review on Basidiospores of lamellate agarics. Mycosphere 6:78–112. doi:10.5943/mycosphere/6/1/10

[8] Money NP. 2016. Spore production, discharge, and dispersal, p 67–97. In The fungi. Elsevier.

[9] Romero F, Cazzato S, Walder F, Vogelgsang S, Bender SF, van der Heijden MGA. 2022. Humidity and high temperature are important for predicting fungal disease outbreaks worldwide. New Phytol 234:1553–1556. doi:10.1111/nph.17340 33713447

[10] Trail F, Xu H, Loranger R, Gadoury D. 2002. Physiological and environmental aspects of ascospore discharge in Gibberella zeae (anamorph Fusarium graminearum). Mycologia 94:181–189. 21156487

[11] Anderson PK, Cunningham AA, Patel NG, Morales FJ, Epstein PR, Daszak P. 2004. Characteristics of the pathogens and drivers of emerging infectious diseases of plants. Trends Ecol Evol (Amsterdam) 10:535–544. doi:10.1016/j.tree.2004.07.021 16701319

[12] Noblin X, Yang S, Dumais J. 2009. Surface tension propulsion of fungal spores. J Exp Biol 212:2835–2843. doi:10.1242/jeb.029975 19684219

[13] Ahimera N, Gisler S, Morgan DP, Michailides TJ. 2004. Effects of single-drop impactions and natural and simulated rains on the dispersal of Botryosphaeria dothidea conidia. Phytopathology 94:1189–1197. doi:10.1094/PHYTO.2004.94.11.1189 18944454

[14] Eskalen A, Faber B, Bianchi M. 2013. Spore trapping and pathogenicity of fungi in the Botryosphaeriaceae and Diaporthaceae associated with avocado branch canker in California. Plant Dis 97:329–332. doi:10.1094/PDIS-03-12-0260-RE 30722352

[15] Tronsmo AM, Tronsmo A, Jørgensen HJL, L-s M. 2020. Plant pathogenic fungi, p 41–74. CABI Wallingford UK.

[16] Oostlander AG, Brodde L, von Bargen M, Leiterholt M, Trautmann D, Enderle R, Elfstrand M, Stenlid J, Fleißner A. 2023. A reliable and simple method for the production of viable pycnidiospores of the pine pathogen Diplodia sapinea and a spore-based infection assay on scots pine. Plant Dis 107:3370–3377. doi:10.1094/PDIS-01-23-0107-RE 37163310

[17] Mehl J, Wingfield MJ, Roux J, Slippers B. 2017. Invasive everywhere? phylogeographic analysis of the globally distributed tree pathogen Lasiodiplodia theobromae. Forests 8:145. doi:10.3390/f8050145

[18] Phillips AJL, Alves A, Abdollahzadeh J, Slippers B, Wingfield MJ, Groenewald JZ, Crous PW. 2013. The Botryosphaeriaceae: genera and species known from culture. Stud Mycol 76:51–167. doi:10.3114/sim0021 24302790 PMC3825232

[19] Avenot HF, Vega D, Arpaia ML, Michailides TJ. 2023. Prevalence, identity, pathogenicity, and infection dynamics of Botryosphaeriaceae causing avocado branch canker in California. Phytopathology 113:1034–1047. doi:10.1094/PHYTO-11-21-0459-R 36510362

[20] Ismail AM, Cirvilleri G, Polizzi G, Crous PW, Groenewald JZ, Lombard L. 2012. Lasiodiplodia species associated with dieback disease of mango (Mangifera indica) in Egypt. Australasian Plant Pathol 41:649–660. doi:10.1007/s13313-012-0163-1

[21] Úrbez-Torres JR, Battany M, Bettiga LJ, Gispert C, McGourty G, Roncoroni J, Smith RJ, Verdegaal P, Gubler WD. 2010. Botryosphaeriaceae species spore-trapping studies in California vineyards. Plant Dis 94:717–724. doi:10.1094/PDIS-94-6-0717 30754317

[22] Huda-Shakirah AR, Mohamed Nor NMI, Zakaria L, Leong Y-H, Mohd MH. 2022. Lasiodiplodia theobromae as a causal pathogen of leaf blight, stem canker, and pod rot of Theobroma cacao in Malaysia. Sci Rep 12:8966. doi:10.1038/s41598-022-13057-9 35624295 PMC9142511

[23] Roberts AJ, Punja ZK. 2022. Pathogenicity of seedborne Alternaria and Stemphylium species and stem-infecting Neofusicoccum and Lasiodiplodia species to cannabis (Cannabis sativa L., marijuana) plants. Can J Plant Pathol 44:250–269. doi:10.1080/07060661.2021.1988712

[24] Gunamalai L, Duanis-Assaf D, Sharir T, Maurer D, Feygenberg O, Sela N, Alkan N. 2023. Comparative characterization of virulent and less-virulent Lasiodiplodia theobromae isolates. Mol Plant Microbe Interact 36:502–515. doi:10.1094/MPMI-11-22-0234-R 37147768

[25] Sharma G, Elazar M, Maymon M, Meshram V, Freeman S. 2024. Identification and pathogenicity of Lasiodiplodia and Neoscytalidium species associated with mango (Mangifera indica) dieback disease in Israel. Phytoparasitica 52:8. doi:10.1007/s12600-024-01123-z

[26] Trail F. 2007. Fungal cannons: explosive spore discharge in the ascomycota. FEMS Microbiol Lett 276:12–18. doi:10.1111/j.1574-6968.2007.00900.x 17784861

[27] Hernández-Hernández D, Siverio de la Rosa F, Grobler C, Slippers B. 2026. Standardised sporulation methods for Diplodia, Lasiodiplodia and Neofusicoccum. IMA Fungus 17:e176189. doi:10.3897/imafungus.17.176189 41695784 PMC12905590

[28] Shulhani R, Shtienberg D. 2018. Aerial dissemination of Lasiodiplodia theobromae and L. pseudotheobromae pycnidiospores. NIBIO Book, Lillehammer.

[29] Mohankumar V, Dann EK, Akinsanmi OA. 2023. Seasonal dynamics of inoculum of Botryosphaeriaceae in macadamia orchards in Australia. Plant Pathol 72:1160–1170. doi:10.1111/ppa.13730

[30] Moral J, Morgan D, Trapero A, Michailides TJ. 2019. Ecology and epidemiology of diseases of nut crops and olives caused by Botryosphaeriaceae fungi in California and Spain. Plant Dis 103:1809–1827. doi:10.1094/PDIS-03-19-0622-FE 31232653

[31] Billones-Baaijens R, Savocchia S. 2019. A review of Botryosphaeriaceae species associated with grapevine trunk diseases in Australia and New Zealand. Australasian Plant Pathol 48:3–18. doi:10.1007/s13313-018-0585-5

[32] Silva-Valderrama I, Úrbez-Torres J-R, Davies TJ. 2024. From host to host: the taxonomic and geographic expansion of Botryosphaeriaceae. Fungal Biol Rev 48:100352. doi:10.1016/j.fbr.2023.100352

[33] Wijayawardene N, Hyde K, Dai D, Sánchez-García M, Goto B, Saxena R, Erdoğdu M, Selçuk F, Rajeshkumar K, Aptroot A, et al.. 2022. Outline of fungi and fungus-like taxa – 2021. Mycosphere 13:53–453. doi:10.5943/mycosphere/13/1/2

[34] Batista E, Lopes A, Alves A. 2021. What do we know about Botryosphaeriaceae? An overview of a worldwide cured dataset. Forests 12:313. doi:10.3390/f12030313

[35] Rodríguez-Gálvez E, Hilário S, Batista E, Lopes A, Alves A. 2021. Lasiodiplodia species associated with dieback of avocado in the coastal area of Peru. Eur J Plant Pathol 161:219–232. doi:10.1007/s10658-021-02317-5

[36] Wang B, Abubakar YS, Wang Z. 2023. Special issue “Genomics of Fungal Plant Pathogens”. J Fungi (Basel) 9:713. doi:10.3390/jof9070713 37504702 PMC10381389

[37] Peng Y, Li SJ, Yan J, Tang Y, Cheng JP, Gao AJ, Yao X, Ruan JJ, Xu BL. 2021. Research progress on phytopathogenic fungi and their role as biocontrol agents. Front Microbiol 12:670135. doi:10.3389/fmicb.2021.670135 34122383 PMC8192705

[38] Mukherjee R, Gruszewski HA, Bilyeu LT, Schmale DG, Boreyko JB. 2021. Synergistic dispersal of plant pathogen spores by jumping-droplet condensation and wind. Proc Natl Acad Sci USA 118. doi:10.1073/pnas.2106938118 PMC8403951 34417298

[39] Pellitier PT, Kling MM, Qin C, Van Nuland ME, Zhu K, Peay KG. 2025. Wind patterns influence the dispersal and assembly of North American soil fungal communities. Ecol Lett 28:e70130. doi:10.1111/ele.70130 40353721

[40] Gougherty AV, Davies TJ. 2022. A global analysis of tree pests and emerging pest threats. Proc Natl Acad Sci USA 119. doi:10.1073/pnas.2113298119 PMC9060442 35312373

[41] Belair M, Restrepo-Leal JD, Praz C, Fontaine F, Rémond C, Fernandez O, Besaury L. 2023. Botryosphaeriaceae gene machinery: correlation between diversity and virulence. Fungal Biol 127:1010–1031. doi:10.1016/j.funbio.2023.03.004 37142361

[42] Liu J-K, Phookamsak R, Doilom M, Wikee S, Li Y-M, Ariyawansha H, Boonmee S, Chomnunti P, Dai D-Q, Bhat JD, Romero AI, Zhuang W-Y, Monkai J, Jones EBG, Chukeatirote E, Ko Ko TW, Zhao Y-C, Wang Y, Hyde KD. 2012. Towards a natural classification of Botryosphaeriales. Fungal Divers 57:149–210. doi:10.1007/s13225-012-0207-4

[43] Antony S, Billones-Baaijens R, Steel CC, Stodart BJ, Savocchia S. 2024. Pathogenicity and progression of Botryosphaeriaceae associated with dieback in walnut orchards in Australia. Eur J Plant Pathol 168:723–742. doi:10.1007/s10658-023-02794-w

[44] Xing Q, Zhou X, Cao Y, Peng J, Zhang W, Wang X, Wu J, Li X, Yan J. 2023. The woody plant-degrading pathogen Lasiodiplodia theobromae effector LtCre1 targets the grapevine sugar-signaling protein VvRHIP1 to suppress host immunity. J Exp Bot 74:2768–2785. doi:10.1093/jxb/erad055 36788641 PMC10112684

[45] Galsurker O, Diskin S, Maurer D, Feygenberg O, Alkan N. 2018. Fruit stem-end rot. Horticulturae 4:50. doi:10.3390/horticulturae4040050 PMC7232454 32295088

[46] Tennakoon D, Phillips A, Phookamsak R, Ariyawansa H, Bahkali A, Hyde K. 2016. Sexual morph of Lasiodiplodia pseudotheobromae (Botryosphaeriaceae, Botryosphaeriales, Dothideomycetes) from China. Mycosphere 7:990–1000. doi:10.5943/mycosphere/si/1b/11

[47] Pusey PL. 1989. Availability and dispersal of ascospores and conidia of Botryosphaeria in peach orchards. Phytopathology 79:635. doi:10.1094/Phyto-79-635

[48] Alves A, Crous PW, Correia A, Phillips A. 2008. Morphological and molecular data reveal cryptic speciation in Lasiodiplodia theobromae. Fungal Divers 28:1–13.

[49] Correia KC, Silva MA, de Morais MA Jr, Armengol J, Phillips AJL, Câmara MPS, Michereff SJ. 2016. Phylogeny, distribution and pathogenicity of Lasiodiplodia species associated with dieback of table grape in the main Brazilian exporting region. Plant Pathol 65:92–103. doi:10.1111/ppa.12388

[50] Phillips AJL, Alves A, Pennycook SR, Johnston PR, Ramaley A, Akulov A, Crous PW. 2008. Resolving the phylogenetic and taxonomic status of dark-spored teleomorph genera in the Botryosphaeriaceae. Persoonia 21:29–55. doi:10.3767/003158508X340742 20396576 PMC2846129

[51] Feygenberg O, Diskin S, Maurer D, Alkan N. 2021. Effect of biological and chemical treatments during flowering on stem-end rot disease, and mango yield. Plant Dis 105:1602–1609. doi:10.1094/PDIS-03-19-0612-RE 33337236

[52] Travadon R, Bousset L, Saint‐Jean S, Brun H, Sache I. 2007. Splash dispersal of Leptosphaeria maculans pycnidiospores and the spread of blackleg on oilseed rape. Plant Pathol 56:595–603. doi:10.1111/j.1365-3059.2007.01572.x

[53] Phillips AJL, Hyde KD, Alves A, Liu J-K (Jack. 2019. Families in Botryosphaeriales: a phylogenetic, morphological and evolutionary perspective. Fungal Divers 94:1–22. doi:10.1007/s13225-018-0416-6

[54] Dinis L-T, Jesus C, Amaral J, Gómez-Cadenas A, Correia B, Alves A, Pinto G. 2022. Water deficit timing differentially affects physiological responses of grapevines infected with Lasiodiplodia theobromae. Plants (Basel) 11:196–212. doi:10.3390/plants11151961 35956441 PMC9370450

[55] Sakalidis ML, Ray JD, Lanoiselet V, Hardy GES, Burgess TI. 2011. Pathogenic Botryosphaeriaceae associated with Mangifera indica in the kimberley region of Western Australia. Eur J Plant Pathol 130:379–391. doi:10.1007/s10658-011-9760-z

[56] Zhang J. 2014. *Lasiodiplodia theobromae* in citrus fruit (diplodia stem-end rot), p 309–335. In Postharvest decay. Elsevier.

[57] Rodríguez-Gálvez E, Guerrero P, Barradas C, Crous PW, Alves A. 2017. Phylogeny and pathogenicity of Lasiodiplodia species associated with dieback of mango in Peru. Fungal Biol 121:452–465. doi:10.1016/j.funbio.2016.06.004 28317545

[58] Li Z, Wang Y-T, Gao L, Wang F, Ye J-L, Li G-H. 2014. Biochemical changes and defence responses during the development of peach gummosis caused by Lasiodiplodia theobromae. Eur J Plant Pathol 138:195–207. doi:10.1007/s10658-013-0322-4

[59] El-Komy MH, Ibrahim YE, Al-Saleh MA. 2023. First report of Lasiodiplodia theobromae causing dieback, a destructive disease on mango trees, in Saudi Arabia. Plant Dis 107:563. doi:10.1094/PDIS-04-22-0812-PDN

[60] Kong Q, Xing SJ, Xie K, Jia ZQ, Li ZL, Liu Y, Xue CL, Shi X, Wang CM, Yuan SY. 2023. First report of Lasiodiplodia theobromae causing stem brown rot of loquat in China . Plant Disease 107:2234. doi:10.1094/PDIS-08-22-1982-PDN

[61] Silva FJA, Dos Santos KM, Santos Rego TJ, Armengol J, Rossi V, Michereff SJ, Gonzalez-Dominguez E. 2018. Temporal conidial dispersal pattern of Botryosphaeriaceae species in table-grape vineyards in northeastern Brazil. Phytopathol Mediterr 57:547–556. doi:10.14601/Phytopathol_Mediterr-24240

[62] Amponsah NT, Jones E, Ridgway HJ, Jaspers MV. 2009. Rainwater dispersal of Botryosphaeria conidia from infected grapevines. N Zeal Plant Protect 62:228–233. doi:10.30843/nzpp.2009.62.4824

[63] Correia MS, Costa J da S. 2005. Dispersão anemófila do fungo Lasiodiplodia theobromae em plantações de coqueiro. Fitopatol Bras 30:150–154. doi:10.1590/S0100-41582005000200008

[64] Beck L, Miles M, Steilage T, Hartmann G. 2006. Urediniospore release and escape from rust-infected soybean fields. National Soybean Rust Symposium, St. Louis, MO. USDA Agricultural Research Service

[65] Lima MA, Blum LEB, Uesugi CH. 2019. Hourly and daily changes on airborne urediniospores of Phakopsora Pachyrhizi. Biosci J 35:126–136. doi:10.14393/BJ-v35n1a2019-39413

[66] Boudrot A, Pico J, Merle I, Granados E, Vílchez S, Tixier P, Filho E de MV, Casanoves F, Tapia A, Allinne C, Rice RA, Avelino J. 2016. Shade effects on the dispersal of airborne hemileia vastatrix uredospores. Phytopathology 106:572–580. doi:10.1094/PHYTO-02-15-0058-R 26828230

[67] Savage D, Barbetti MJ, MacLeod WJ, Salam MU, Renton M. 2012. Seasonal and diurnal patterns of spore release can significantly affect the proportion of spores expected to undergo long-distance dispersal. Microb Ecol 63:578–585. doi:10.1007/s00248-011-9949-x 21968611

[68] Wang X, Feng H. 2023. Investigating the role played by osmotic pressure difference in osmotic dehydration: interactions between apple slices and binary and multi-component osmotic systems. Foods 12:3179. doi:10.3390/foods12173179 37685112 PMC10486890

[69] Elbert W, Taylor PE, Andreae MO, Pöschl U. 2007. Contribution of fungi to primary biogenic aerosols in the atmosphere: wet and dry discharged spores, carbohydrates, and inorganic ions. Atmos Chem Phys 7:4569–4588. doi:10.5194/acp-7-4569-2007

[70] Bechinger C, Giebel K-F, Schnell M, Leiderer P, Deising HB, Bastmeyer M. 1999. Optical measurements of invasive forces exerted by appressoria of a plant pathogenic fungus. Science 285:1896–1899. doi:10.1126/science.285.5435.1896 10489364

[71] Loehrer M, Botterweck J, Jahnke J, Mahlmann DM, Gaetgens J, Oldiges M, Horbach R, Deising H, Schaffrath U. 2014. In vivo assessment by Mach-Zehnder double-beam interferometry of the invasive force exerted by the Asian soybean rust fungus (Phakopsora pachyrhizi). New Phytol 203:620–631. doi:10.1111/nph.12784 24725259

[72] Deising HB, Werner S, Wernitz M. 2000. The role of fungal appressoria in plant infection. Microbes Infect 2:1631–1641. doi:10.1016/s1286-4579(00)01319-8 11113382

[73] de Jong JC, McCormack BJ, Smirnoff N, Talbot NJ. 1997. Glycerol generates turgor in rice blast. Nature 389:244–244. doi:10.1038/38418

[74] Foster AJ, Ryder LS, Kershaw MJ, Talbot NJ. 2017. The role of glycerol in the pathogenic lifestyle of the rice blast fungus Magnaporthe oryzae. Environ Microbiol 19:1008–1016. doi:10.1111/1462-2920.13688 28165657

[75] Wang L, Lai Y, Chen J, Cao X, Zheng W, Dong L, Zheng Y, Li F, Wei G, Wang S. 2023. The ASH1–PEX16 regulatory pathway controls peroxisome biogenesis for appressorium-mediated insect infection by a fungal pathogen. Proc Natl Acad Sci USA 120. doi:10.1073/pnas.2217145120 PMC9942893 36649415

[76] Latijnhouwers M, de Wit P, Govers F. 2003. Oomycetes and fungi: similar weaponry to attack plants. Trends Microbiol 11:462–469. doi:10.1016/j.tim.2003.08.002 14557029

[77] Trail F, Gaffoor I, Vogel S. 2005. Ejection mechanics and trajectory of the ascospores of Gibberella zeae (anamorph Fuarium graminearum). Fungal Genet Biol 42:528–533. doi:10.1016/j.fgb.2005.03.008 15878295

[78] Fischer M, Cox J, Davis DJ, Wagner A, Taylor R, Huerta AJ, Money NP. 2004. New information on the mechanism of forcible ascospore discharge from Ascobolus immersus. Fungal Genet Biol 41:698–707. doi:10.1016/j.fgb.2004.03.005 15275665

[79] Winston PW, Bates DH. 1960. Saturated solutions for the control of humidity in biological research. Ecology 41:232–237. doi:10.2307/1931961

[80] Singh SP, Gaur R. 2017. Endophytic Streptomyces spp. underscore induction of defense regulatory genes and confers resistance against Sclerotium rolfsii in chickpea. Biol Control 104:44–56. doi:10.1016/j.biocontrol.2016.10.011

[81] Lisec J, Schauer N, Kopka J, Willmitzer L, Fernie AR. 2006. Gas chromatography mass spectrometry-based metabolite profiling in plants. Nat Protoc 1:387–396. doi:10.1038/nprot.2006.59 17406261

