# Supplementary material for: A novel mechanism behind aerial dispersal of pycnidiospores
Source: mSphere. 2026 Jun 10;11(6):e00034-26. doi: 10.1128/msphere.00034-26 (PMC13317220; doi:10.1128/msphere.00034-26)
Supplement: Supplemental figures — Fig. S1 to S7. [file msphere.00034-26-s0001.docx]

**Supplementary Materials**

**A novel mechanism behind aerial dispersal of pycnidiospores**

Satyendra Pratap Singh^1^, Alon Shomron^1,3^, Ran Shulhani^2^, Dani Shtienberg^2^, and Noam Alkan^1^*

^1^Department of Postharvest Science, ARO, Volcani Center, PO Box 15159 68 HaMakkabbim Rd., Rishon LeZiyyon 7528809. Israel

^2^Department of Plant Pathology and Weed Science, ARO, Volcani Center, PO Box 15159 68 HaMakkabbim Rd., Rishon LeZiyyon 7528809. Israel

^3^Faculty of Agriculture, Food and Environment, The Hebrew University of Jerusalem, Rehovot 76100, Israel.

***Corresponding Author:** Dr. Noam Alkan, Department of Postharvest Science, ARO, the Volcani Center, Rishon LeZiyyon 7528809. Israel. Email: [noamal@volcani.agri.gov.il](mailto:noamal@volcani.agri.gov.il)


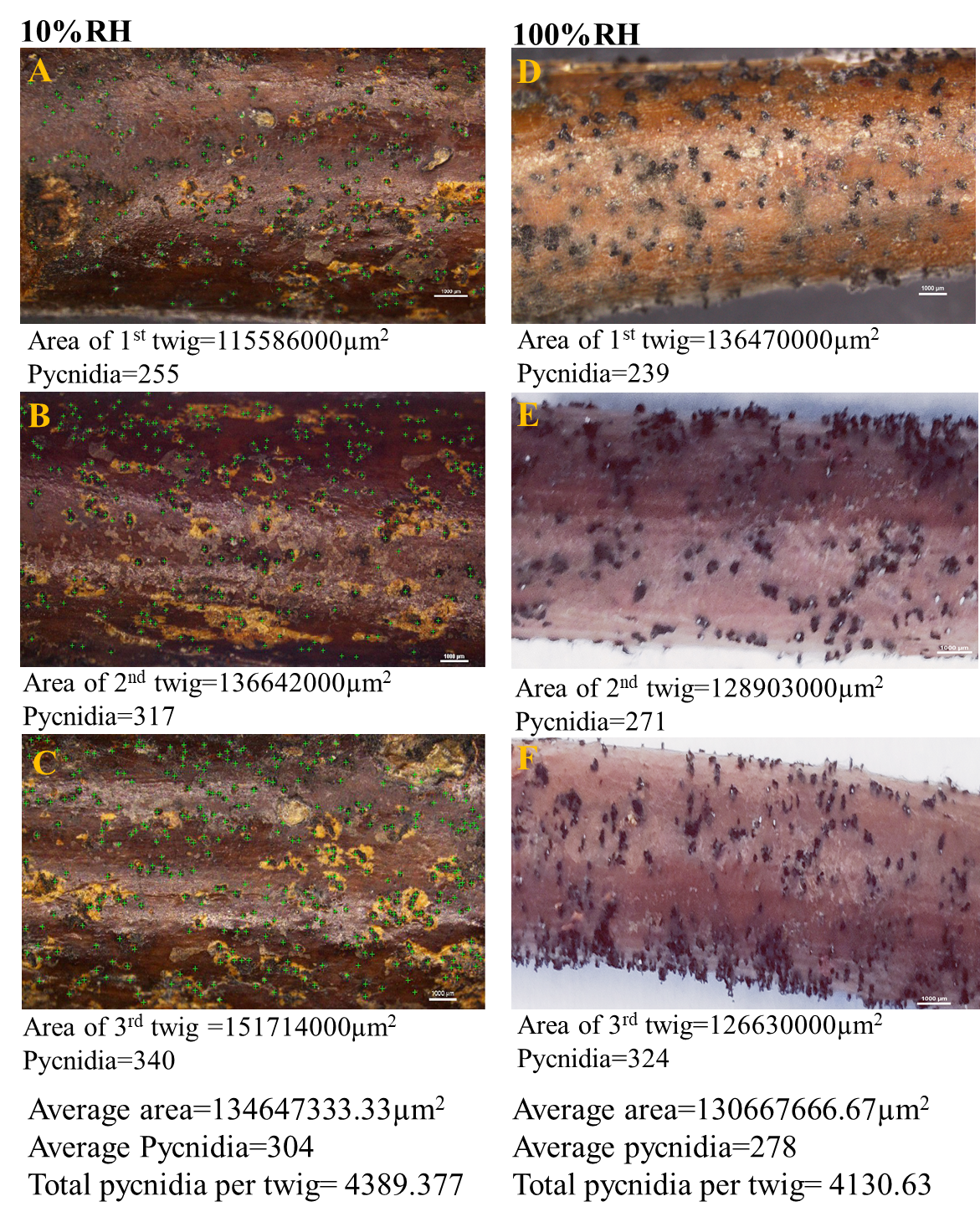


**Figure S1.** Assessment of pycnidia formation on infected twigs at 10% (A-C) and 100% (D-F) RH conditions. Green markers (+), added to the left-hand images using NIS-Elements BR (version 5.02.03) software integrated with an Olympus compound microscope, were used to quantify the total number of pycnidia formed on the infected twig.

**
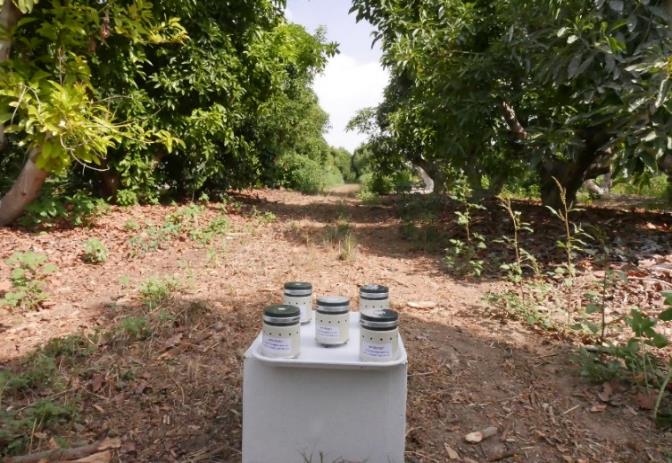
**

**Figure S2.** Arrangement of different Burkard spore traps to assess the pycnidiospores count in an avocado orchard.


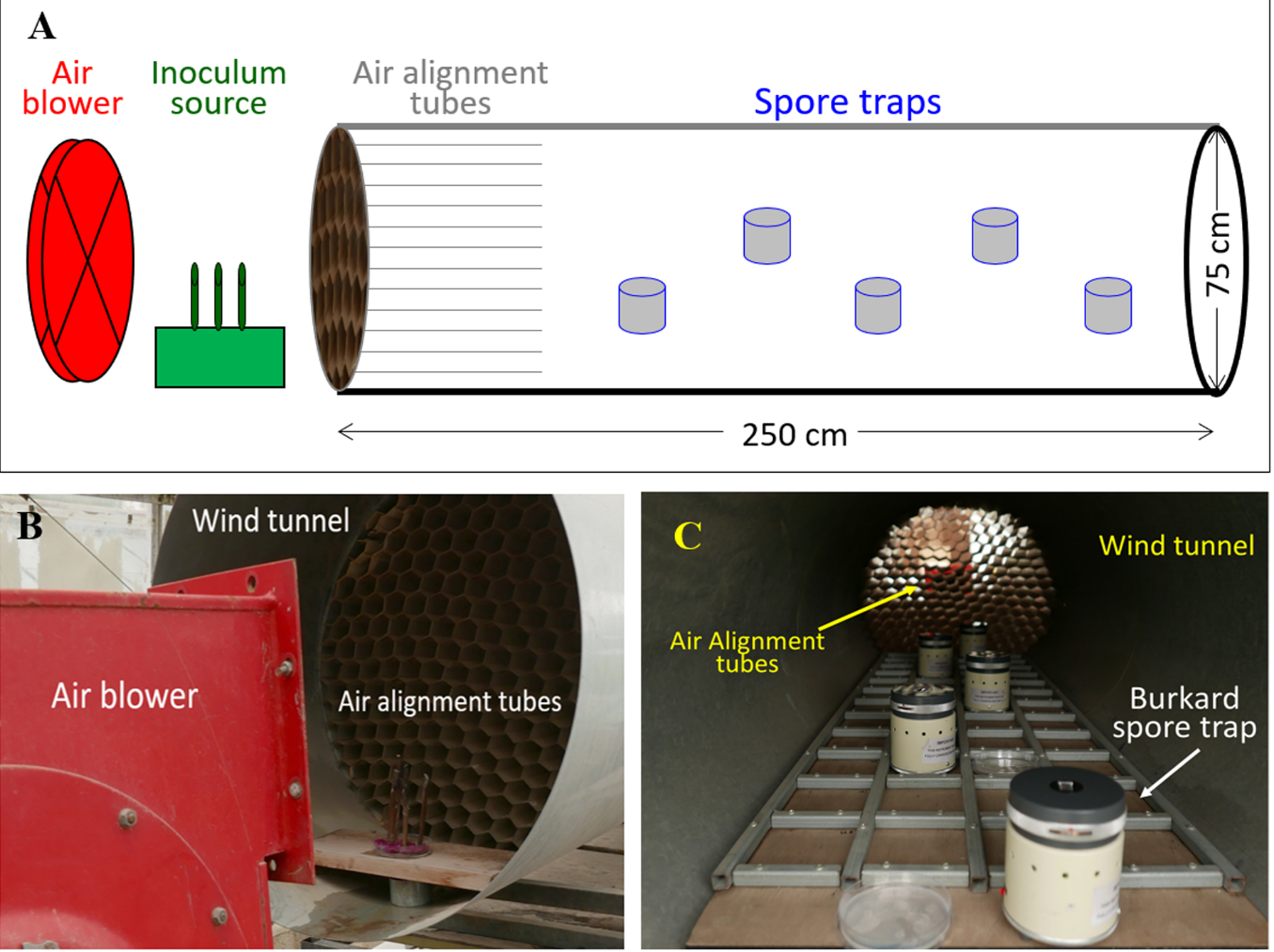


**Figure S3.** Set up a wind tunnel for *in vivo* experiments. **(A)** Schematic diagram of the experimental set-up. **(B)** Alignment of infected twigs and an air blower for the execution of an *in vitro* experiment by using a wind tunnel. **(C&D)** Arrangement of different Burkard spore traps to assess the pycnidiospores count in a wind tunnel and an avocado orchard.


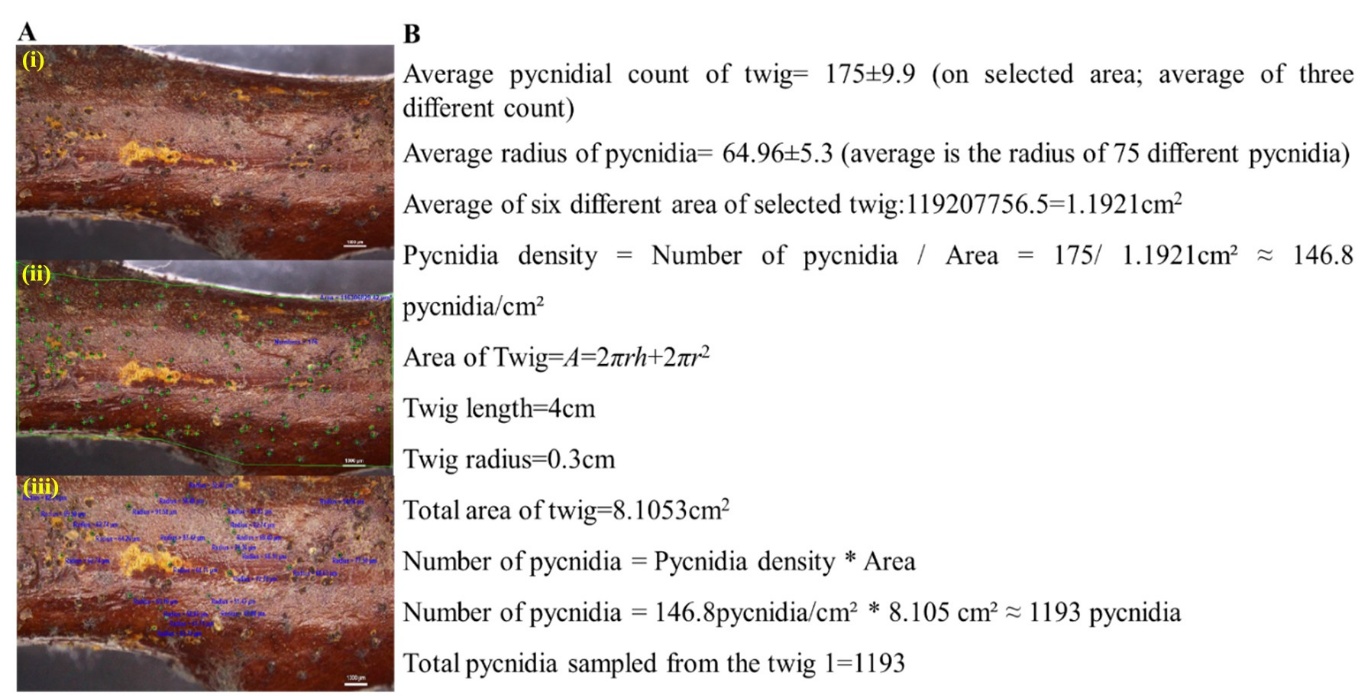


**Figure S4.** Sampling of closed pycnidia from infected twigs. (A) A representative infected twig used for analysis: (i) original image of the selected twig, (ii) selected region of the same twig outlined in green, indicating the analyzed area, with green “+” symbols marking individual pycnidia, and (iii) measurement of pycnidia size, where blue markings denote the radius of individual pycnidia. (B) Quantification of pycnidia, including counts obtained from the selected twig area.


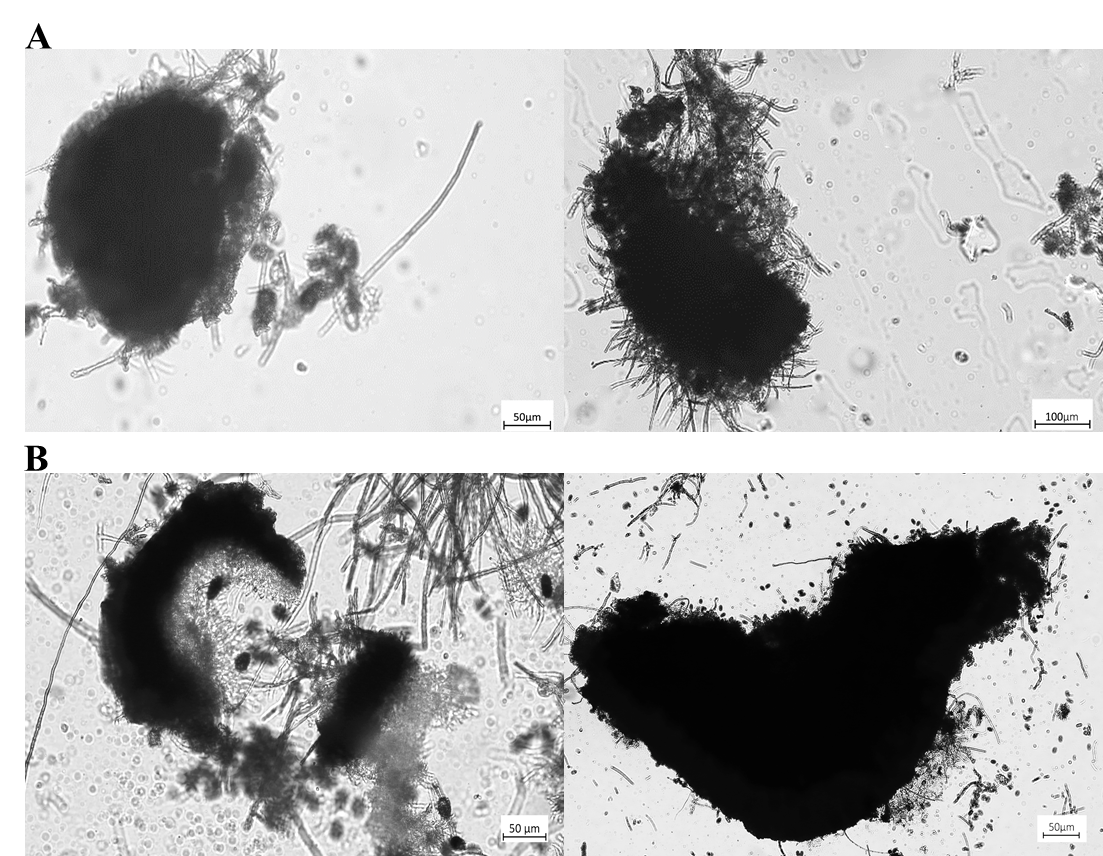


**Figure S5.** Micrographs of sampled pycnidia. **(A)** Matured closed pycnidia before the supplementation of sterilized water. **(B)** Burst pycnidia after the supplementation of sterilized water.

**A**


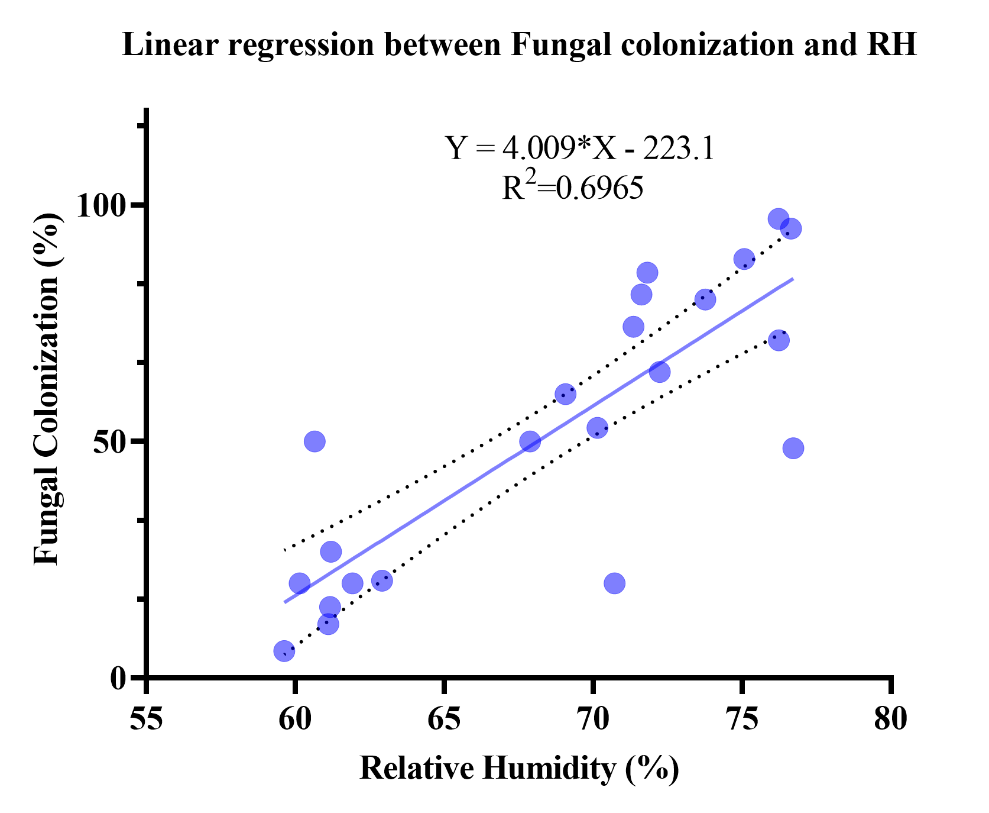


**B**


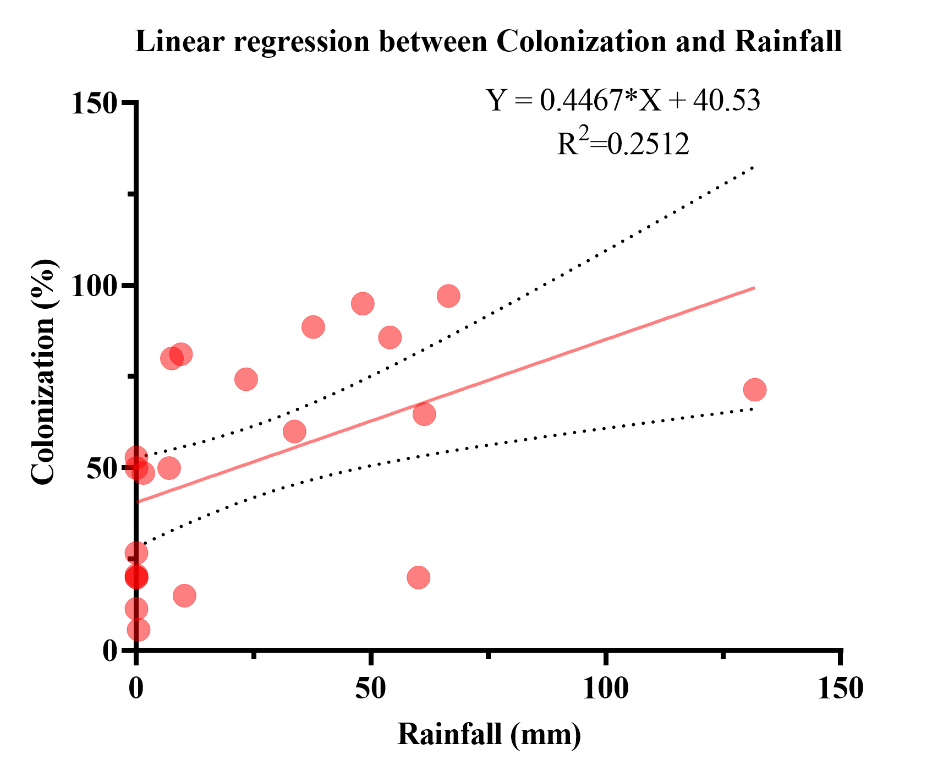


**Figure S6:** Linear regression analysis between **(A)** *Lasiodiplodia* colonization incidence in young green twigs of avocado plantation and relative humidity (%) of respective years. **(B)** *Lasiodiplodia* colonization incidence in young green twigs of avocado plantation and monthly rainfall (mm) of respective years. Colonization pattern was examined over three years at 22 time points, with 33-35 twigs assessed at each time point. Monthly relative humidity and rainfall data of respective years were obtained from the Israeli Meteorological Service (IMS).


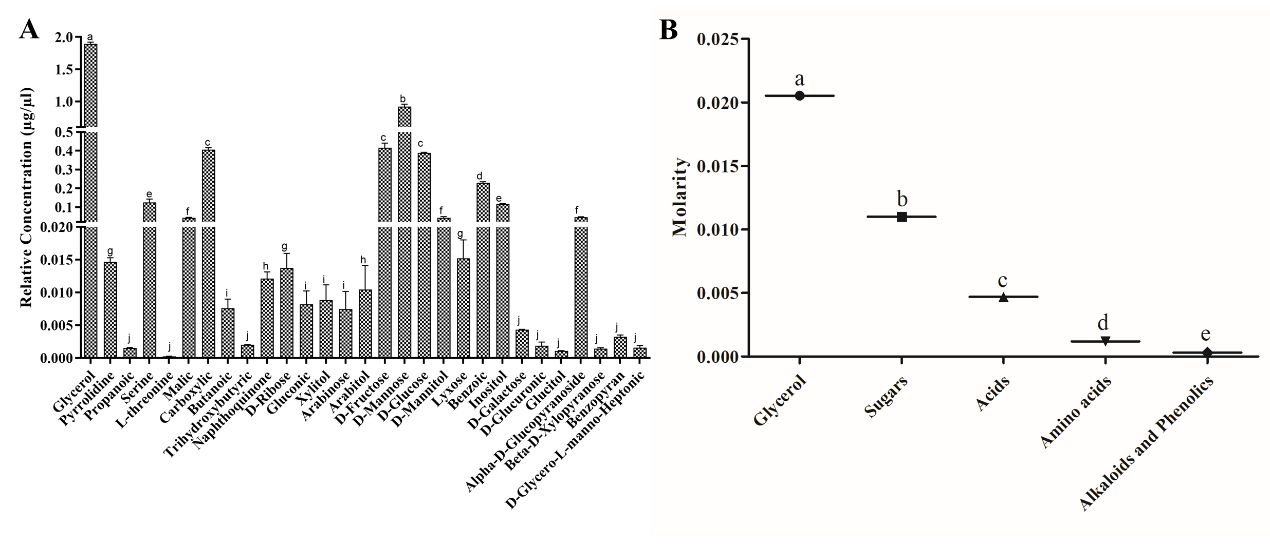

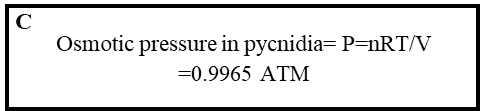


**­­­Figure S7.** GC-MS Profiling of pycnidial sap from open pycnidia **(A)** Relative concentration of different compounds of pycnidial sap; **(B)** Different groups found in pycnidial sap; **(C)** Calculated osmotic pressure (ATM) in pycnidia based on the molarity of different compounds. Values are the mean of three replicates with standard error (SE) indicated. Means followed by the letter(s) on bars indicate significant differences according to Tukey’s multiple comparison tests (P ≤ 0.05).
